# Mental health and healthcare use during COVID‐19 among neurodivergent youth and youth with pre‐existing psychiatric disorders: A systematic review

**DOI:** 10.1002/jcv2.70176

**Published:** 2026-10-08

**Authors:** Ulyana Sirotina, Bryndis Lara Bjarnadottir, Agnieszka Butwicka, Eivind Ystrom, Jaana Suvisaari, Olli Kiviruusu, Audun Valborgarson, Heiddis Bjork Valdimarsdottir, Helga Ask, Unnur Valdimarsdottir, Thorhildur Halldorsdottir

**Affiliations:** ^1^ Department of Psychology Reykjavik University Reykjavik Iceland; ^2^ Department of Medical Epidemiology and Biostatistics Karolinska Institute Stockholm Sweden; ^3^ Division of Mental Health Services R&D Department Akershus University Hospital Lørenskog Norway; ^4^ Institute of Clinical Medicine Faculty of Medicine University of Oslo Oslo Norway; ^5^ Department of Biostatistics and Translational Medicine Medical University of Lodz Lodz Poland; ^6^ PROMENTA Research Center Department of Psychology Faculty of Social Sciences University of Oslo Oslo Norway; ^7^ Department of Child Health and Development Norwegian Institute of Public Health Oslo Norway; ^8^ Department of Healthcare and Social Welfare Finnish Institute for Health and Welfare Helsinki Finland; ^9^ Department of Population Health Science and Policy Icahn School of Medicine at Mount Sinai New York New York USA; ^10^ PsychGen Centre Norwegian Institute of Public Health Oslo Norway; ^11^ Centre of Public Health Sciences Faculty of Medicine School of Health Sciences University of Iceland Reykjavik Iceland; ^12^ Department of Epidemiology Harvard T.H. Chan School of Public Health Boston Massachusetts USA; ^13^ Department of Physiology and Pharmacology Karolinska Institute Stockholm Sweden

**Keywords:** COVID‐19, mental health, neurodivergent youth, psychiatric disorders, psychiatric healthcare, youth

## Abstract

**Background:**

Neurodivergent youth and youth with pre‐existing psychiatric disorders may have faced additional challenges in navigating the demands and restrictions of the COVID‐19 pandemic. However, less is known about how mental health outcomes and psychiatric healthcare utilization differed across diagnostic groups and other youth‐related factors. This systematic review synthesizes evidence on mental health symptoms and psychiatric healthcare use among youth with pre‐existing conditions during the pandemic.

**Methods:**

We searched PubMed, Web of Science and additional sources for cross‐sectional and longitudinal studies with data collected between March 2020 and May 2023. Eligible studies included neurodivergent youth and/or youth with psychiatric disorders confirmed through clinician diagnosis or medical records prior to the pandemic. Given the clinical and methodological diversity of studies, findings were synthesized narratively.

**Results:**

Forty‐eight studies met the inclusion criteria. Mental health outcomes showed mixed patterns both across and within diagnostic groups, with individual studies reporting symptom increase, stability and improvements. Evidence on psychiatric healthcare utilization was more limited and suggested different patterns between service use and psychotropic medication use. Compared to boys, girls were more likely to experience COVID‐related distress and increases in psychiatric service use during the pandemic. Age‐related findings varied across developmental stages and outcome domains. Most studies (*n* = 40; 83.3%) had a moderate overall risk of bias (RoB), while seven (14.6%) had a high overall RoB.

**Conclusion:**

Mental health outcomes among youth with pre‐existing conditions during the COVID‐19 pandemic varied both within and across diagnostic groups. Outcomes were also associated with individual, developmental, contextual and service‐related factors. These findings suggest the importance of considering such factors alongside diagnosis when identifying clinical needs and planning support during large‐scale societal disruptions. Evidence on psychiatric healthcare utilization remains limited. More methodologically robust research is needed to clarify mental health and healthcare‐use patterns during major public health emergencies.

## Key Points

**What's known?**

- Neurodivergent youth and youth with pre‐existing psychiatric disorders were considered particularly vulnerable to mental health challenges during the COVID‐19 pandemic. However, emerging evidence indicated that the findings were mixed.

**What's new?**

- Mental health outcomes varied both across and within pre‐existing conditions, with individual studies reporting worsening, stability or improvement.
- Outcomes were also associated with gender, age, comorbid conditions, emotion regulation, pre‐pandemic symptom severity, irritability, exposure to pandemic‐related stress and access to care.

**What's relevant?**

- Youth outcomes varied within as well as across diagnostic groups and were also associated with individual, developmental, contextual and service‐related factors.
- These findings suggest the importance of considering these factors alongside diagnosis when identifying clinical needs and tailoring support.

## INTRODUCTION

The COVID‐19 pandemic caused widespread daily life disruptions for children and adolescents worldwide, with fear of contagion, school closures, physical distancing and service interruptions contributing to marked psychosocial stress. Accumulating research indicates that a global increase in mental health problems among children and adolescents (Gyllenberg et al., 2018) was exacerbated during the COVID‐19 pandemic (Madigan et al., 2023). Emerging findings further suggest that these outcomes have not returned to pre‐COVID levels even after the end of the acute pandemic phases (Haskell et al., 2025; Kiviruusu et al., 2024). Thus, COVID‐19 continues to have implications for mental health services and policy development. Gender differences in youth mental health are consistently reported across studies, both before and during the pandemic (Madigan et al., 2023). However, further investigation of vulnerable subgroups is needed to identify their post‐pandemic needs, to guide targeted support and resource allocation and to inform clinical and policy responses in case of future large‐scale societal stressors (Holmes et al., 2020).

Youth who rely on mental health services may have been disproportionately affected by the pandemic‐related interruptions in access and continuity of care. Thus, among those at potentially elevated risk are neurodivergent youth and youth with pre‐existing psychiatric disorders. In this review, neurodivergent youth refers to autistic youth and those with attention‐deficit/hyperactivity disorder (ADHD) or intellectual disability (ID; Botha et al., 2023; Srinivasan, 2025). Psychiatric disorders refer to a range of mental health conditions, including mood, anxiety, conduct, obsessive‐compulsive, eating and somatic symptom disorders. To address both groups at the same time and to indicate the presence of the status before the pandemic, we use the term “youth with pre‐existing conditions.” Emerging research indicates that youth with pre‐existing conditions experienced increased externalizing and internalizing symptoms and used psychotropic medication more often during the pandemic compared to pre‐pandemic years (Bliddal et al., 2023; Sicouri et al., 2023). Externalizing symptoms are understood as outward‐focused problems including aggression, rule‐breaking and attention difficulties, while internalizing symptoms include anxiety, low mood, withdrawal and somatic complaints (Øien et al., 2025).

A recent systematic review and meta‐analysis based on 21 longitudinal studies (*N* = 2617) of youth with pre‐existing conditions (≤18 years) yielded mixed results (Ching et al., 2025). While pooled analyses did not show significant changes in internalizing or externalizing symptoms compared to pre‐pandemic levels, there was substantial variability in the direction of changes across studies. Their narrative analysis suggested that pandemic‐related changes in mental health symptoms could have varied by pre‐existing condition, gender and age. Overall, their findings underscored the complexity of pandemic effects and highlighted the need for broadening the scope of age ranges, follow‐up periods, outcomes and their potential moderators, in order to capture the full range of pandemic‐related effects in clinical populations.

Building on previous work (Ching et al., 2025), this systematic review takes a broader perspective on the impact of the COVID‐19 pandemic among youth with pre‐existing conditions. We include both longitudinal and cross‐sectional studies, extend the age range up to 24 years (Sawyer et al., 2018) and consider psychiatric healthcare utilization alongside mental health symptoms. We aimed to specifically examine how outcomes varied across diagnostic groups and other youth‐related factors. Given this broader scope and the resulting clinical and methodological diversity of the evidence, we used a narrative synthesis to integrate findings across study designs and outcomes. This approach allows us to identify clinically relevant patterns of vulnerability and resilience, highlight areas in which evidence remains limited and identify priorities for mental health services and future research. The main questions of this review were:

1. How did mental health symptoms and psychiatric healthcare utilization during the COVID‐19 pandemic vary across pre‐existing conditions?
2. Which other youth‐related factors were associated with their outcomes?

## METHODS

Given the clinical and methodological diversity among eligible studies (Baumeister, 2013), we decided to conduct a narrative review and not a meta‐analysis.

### Eligibility criteria

Eligible studies included children and adolescents (≤24) with a neurodivergence status or psychiatric diagnosis prior to the COVID‐19 pandemic. The condition must have been assigned by a healthcare professional and/or documented in the registry data. Pre‐pandemic use of psychotropic medication was also accepted as a proxy for diagnosis.

This review included studies that examined mental health symptoms (e.g., depressive symptoms), mental health service use (e.g., mental health consultations) or psychotropic medication use (e.g., prescriptions) among youth with pre‐existing conditions during the pandemic. These outcomes could be compared over time (during vs. before COVID‐19) or across groups (e.g., contrasting specific diagnostic groups or youth with vs. without pre‐existing conditions).

Quantitative observational designs, including cross‐sectional and longitudinal studies, were included. Mixed methods studies could be included if they reported relevant quantitative data, whereas interventional and qualitative‐only studies were excluded. In each study, at least some data was collected between March 2020 (pandemic onset; World Health Organization, 2020) and May 2023 (end of the pandemic; World Health Organization, 2023). While only studies published in English were included, research conducted in any country was eligible.

### Identification process

A comprehensive search began on March 28, 2025 and ended on September 14, 2026. PubMed and Web of Science were the main information sources of this review (see search terms in Table 1). Additional methods of identifying studies included a thorough examination of studies in existing systematic reviews (Chen et al., 2025; Ching et al., 2025; Dal Pai et al., 2024; Kauhanen et al., 2023; Luginaah et al., 2023, 2025; Madigan et al., 2023, 2025; Orban et al., 2024; Panda et al., 2021; Rogers & MacLean, 2023; Wan Mohd Yunus et al., 2022), searching repositories and pre‐print servers, such as PsychArchives and bioRxiv, contacting experts and reviewing the reference lists of papers included in this review.

**TABLE 1:** Search terms used on PubMed and Web of Science.

Concept	Search term
Exposure	COVID^a^
	AND
Participants	(child* OR adolesc* OR pediatric OR youth OR teen*)^a^ AND (mental health disorder OR psychiatric disorder OR neurodevelopmental disorder OR depr* OR anxi* OR ADHD OR autis* OR ASD OR “intellectual disability” OR dysthymi* OR phobi* OR “conduct disorder” OR “eating disorder” OR anorexi* OR bulimi* OR bing* OR “obsessive‐compulsive disorder” OR OCD OR “oppositional defiant disorder”) AND (diagnosis OR pre‐existing)

*Note*: Full search strings used on PubMed and Web of Science are available in Supporting Information S1.

^a^ These search terms were applied to the title; all other search terms were not restricted.

Two reviewers (U.S. and B.L.B.) independently assessed the titles and abstracts obtained through the search, applying the criteria. Full‐text reports were retrieved for studies that appeared to meet the criteria or where eligibility was uncertain. The same reviewers then independently evaluated the full‐text articles to determine if they fulfilled all inclusion requirements, with any disagreements (*n* = 3; 1.1%) addressed through discussion with a third reviewer (T.H.).

### Data extraction and synthesis

The information extracted from each study included authors, country, participants' gender and age, sample size, data collection period, study design, participants' pre‐existing conditions, methods of assessing conditions and outcomes (Table 2), as well as main relevant findings (Supporting Information S1: Table S1). Data were extracted by two reviewers (U.S. and B.L.B.), who reviewed both the main articles and relevant supplementary materials. Discrepancies were resolved through discussion, with consultation of a third reviewer (T.H.) when necessary.

Findings were synthesized in a qualitative way. In the first part of our synthesis, studies were grouped based on the type (neurodivergences/psychiatric disorders) and then the number (single/multiple) of examined conditions. Specifically, we started by summarizing findings from studies focused on neurodivergent youth, including studies that focused on a single neurodivergent group and studies that compared multiple neurodivergent groups to each other. Next, we moved to studies which focused on a single pre‐existing psychiatric disorder and then, studies which examined multiple pre‐existing conditions. Within each section, we synthesized evidence across mental health symptoms, mental health service use and psychotropic medication use, while examining potential moderating factors for each outcome. In the second part of the synthesis, findings were summarized across all studies to enable comparisons between youth with pre‐existing conditions and their peers without such conditions. A summary on potential moderating factors across studies is available in Supporting Information S1 (Table S2).

### Risk of bias

All included studies were assessed for the risk of bias (RoB). Our RoB assessment was based on the procedure described by Ching et al. (2025), adapted to account for the inclusion of cross‐sectional studies. Five indicators were used to determine the overall RoB: recruitment/sampling strategy, sample size adequacy, outcome assessment, attrition/response rate and control for confounding (see further details in Supporting Information S1).

A calibration‐based approach (Bourke et al., 2025; Fortnum et al., 2026) was used for the RoB assessment. To evaluate consistency in the application of the RoB criteria, 16 of the 48 included studies (33.3%) were independently assessed by two reviewers (U.S. and B.L.B.). The reviewers agreed on ratings for 14 of the 16 studies (87.5%), with disagreements in the remaining two studies resolved through discussion with a third reviewer (T.H.). Following this calibration process, the remaining studies were equally divided and assessed by one of the two reviewers.

## RESULTS

A total of 48 studies met inclusion criteria for this systematic review (Figure 1 for a PRISMA flow diagram). With a total approximate sample of 956,162 youth with pre‐existing conditions included in the review, sample sizes of individual studies varied substantially, ranging from 15 (Henein et al., 2024) to around 898,805 (Lund et al., 2025).

**FIGURE 1:**
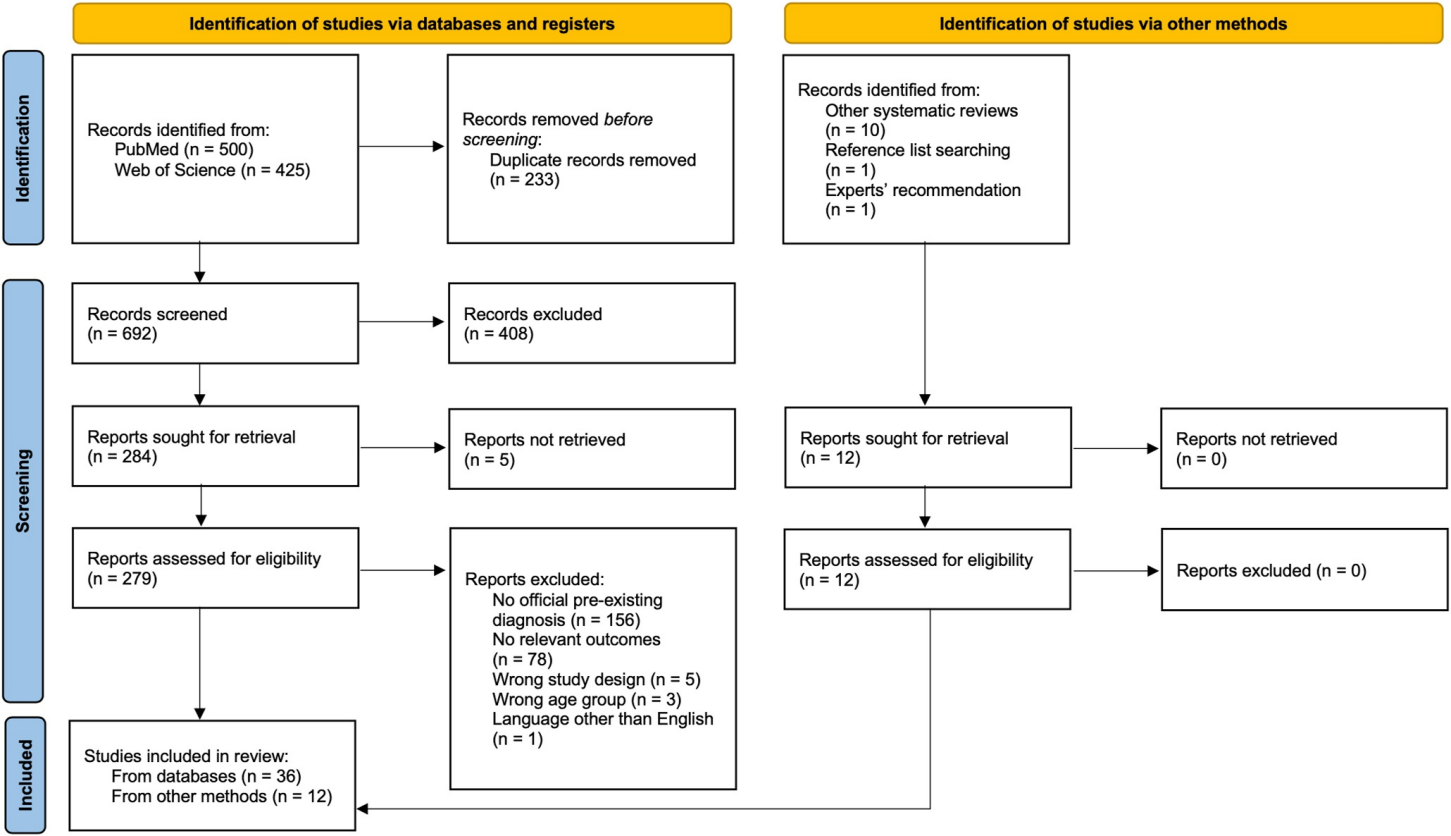
PRISMA flow diagram illustrating study selection procedure. *Source*: Page et al. (2021).

### Study characteristics

Of the included studies, 30 (62.5%) had a longitudinal design (including two with retrospective recall), and 18 (37.5%) were cross‐sectional (Table 2). Regions where the data were collected and pre‐existing conditions that the studies focused on are described in Figure 2. Data collection periods, youth age ranges and gender ratios are documented in Table 2 and visualized in Figure 3.

**TABLE 2:** Description of studies (*N* = 48) included in the systematic review and overall risk of bias ratings.

Authors, country	Gender ratio^a^	Age	Sample size	Data collection period	Study design	Pre‐existing condition	Assessment of pre‐existing condition	Assessment of outcome(s)	Overall risk of bias^b^
Studies focusing on a single ND group									
Berard et al. (2021) France	20.5% female	2–21	239	April 27–May 13, 2020	Cross‐sectional	Autism	Standardized instruments (ADOS‐2, ADI‐R, VABS‐II) administered by a clinician	Clinician‐rated: ADOS‐2 and VABS‐II Parent‐report: COVID survey questions	Moderate
Charalampopoulou et al. (2022) Canada	24.2% female	6–18	265	Pre‐pandemic (“the latest time point before lockdown”) and during the pandemic (May–December 2020)	Longitudinal	Autism	Standardized instruments (ADOS‐2, ADI‐R) administered by a clinician	Parent‐report: RCADS, TOCS, TIDES, SWAN and survey questions Parent‐report and self‐report: CRISIS_AFAR	Moderate
Day et al. (2023) USA	20% female (ND) 56% female (NT)	8.5–17.4	51 (ND) 25 (NT)	June–December 2020	Longitudinal	Autism	Standardized instrument (ADOS‐2) administered by a clinician	Parent‐report and self‐report: RCADS	Moderate
de Maat et al. (2025) Netherlands	25.8% female (ND) 24.2% female (NT)	4–21	62 (ND) 213 (NT)	Pre‐pandemic (March 2019–March 2020 for the ND; January 2018–January 2020 for the NT) During the pandemic (January–May 2021 for the ND; December 2020–March 2021 for the NT)	Longitudinal	Autism	Clinical assessment	Parent‐report BPM for the ND Self‐report BPM for the NT	Moderate
Gonzales et al. (2023) USA	22.3% female	1–14	283	Pre‐pandemic (March 2019–February 2020) and during the pandemic (March–May 2020; June 2020–February 2021)	Longitudinal	Autism	Register‐based	Register‐based	Moderate
Hooijman et al. (2024) Netherlands	34.6% female	4–21	26	Pre‐pandemic (March 1, 2019–March 1, 2020) and during the pandemic (1 year and 1.5 years into the pandemic)	Longitudinal	Autism	Clinical assessment	Parent‐report: SRS‐2	Moderate
Lugo‐Marín et al. (2021) Spain	13.5% female	3–17	37	Pre‐pandemic (unspecified) and during the pandemic (8 weeks after the first lockdown)	Longitudinal	Autism	Clinical assessment	Parent‐report: CBCL and survey questions	High
Mpaka et al. (2026) Democratic Republic of Congo	16.2% female	3–17	68	April 15–July 15, 2020	Cross‐sectional	Autism	Standardized instrument (CARS) administered by a clinician	Parent‐report: Survey questions	Moderate
Palmer et al. (2023) England	20.5% female	15.9–20.1	112	Pre‐pandemic (unspecified) and during the pandemic (July–October 2020)	Longitudinal	Autism	Multispecialist clinical assessment and standardized instruments (e.g., ADOS‐2, ADI‐R)	Parent‐report: SCQ, SDQ and survey questions	Moderate
Siracusano et al. (2021) Italy	24.2% female (preschool group) 17.3% female (school group)	2–5 6–18	33 (preschool group) 52 (school group)	Pre‐pandemic (2019) and during the pandemic (May–July 2020)	Longitudinal	Autism	Multispecialist clinical assessment and standardized instrument (ADOS‐2) administered by a clinician	Parent‐report: ABAS‐II, RBS‐R, CBCL	Moderate
Taniguchi et al. (2022) USA	18.5% female (visited the clinic only in 2019) 20.7% female (visited the clinic in 2019 and 2020)	3–21	378 (visited the clinic only in 2019) 473 (visited the clinic in 2019 and 2020)	Pre‐pandemic (January–December 2019) and during the pandemic (until December 2020)	Longitudinal	Autism	Register‐based	Register‐based	Moderate
Türkoğlu et al. (2021) Turkey	17.4% female	4–17	46	Pre‐pandemic (January 1–February 29, 2020) and during the pandemic (June 3–17, 2020)	Longitudinal	Autism	Clinical assessment	Parent‐report: ARI, AuBC	High
Vasa et al. (2021) USA	19.5% female	2–16	257	May–June 2020	Cross‐sectional	Autism	Clinical assessment	Parent‐report: Survey questions	Moderate
Breaux et al. (2021) USA	∼44.5% female	15–17	118 (with ADHD) 120 (without ADHD)	Pre‐pandemic (September 2018–February 2020) and during the pandemic (May 15–June 14, 2020; July 1–August 5, 2020)	Longitudinal	ADHD	Standardized instrument (ChIPS) administered under clinician's supervision	Parent‐report: VADRS Self‐report: RCADS, DERS‐SF	Moderate
Çetin et al. (2020) Turkey	30.3% female	8–12	76	May 7–May 14, 2020	Cross‐sectional	ADHD	Register‐based	Parent‐report: T‐DSM‐IV‐S Self‐report: CRIES	High
Cunniff et al. (2024) USA	23.7% female	8–18	396	Pre‐pandemic (January 2019–March 2020) and during the pandemic (April 2020–June 2022)	Longitudinal	ADHD	Register‐based	Register‐based	Moderate
Kuygun Karci and Arici Gurbuz (2022) Turkey	25% female	7–17	100	During the pandemic (unspecified)	Longitudinal (with a retrospective recall)	ADHD	Standardized instrument (K‐SADS) administered by a clinician	Parent‐report: CBCL, CPRS, SNAP‐IV and survey questions	High
Melegari et al. (2021) Italy	14.6% female	5–17	992	June 4–21, 2020	Longitudinal (with a retrospective recall)	ADHD	Clinical assessment	Parent‐report: CBCL	Moderate
Uçar et al. (2022) Turkey	21.4% female	10–12	42	May 7–14, 2020	Cross‐sectional	ADHD	Clinical assessment	Parent‐report: T‐DSM‐IV‐S, RCADS Self‐report: CRIES Parent‐report and self‐report: ARI	Moderate
Studies focusing on multiple ND groups									
Suzuki and Hiratani (2021) Japan	17.5% female	6–16	143	Pre‐pandemic (April 2018–February 2020) and during the pandemic (October–December 2020)	Longitudinal	A range of neurodivergences, including ADHD, autism and ID	Clinical assessment	Parent‐report: ABC‐J	Moderate
Studies focusing on a single pre‐existing psychiatric disorder									
Henein et al. (2024) England	∼51.7% female	8–17	15	Pre‐pandemic (March–October 2018–2019) and during the pandemic (March–October 2020)	Longitudinal	OCD	Register‐based	Clinician‐rated: Progress during the pandemic, based on HoNOSCA scores	Moderate
Nissen et al. (2020) Denmark	63.1% female	7–21	65	April–May 2020	Cross‐sectional	OCD	Standardized instruments (K‐SADS, CYBOCS) administered by a clinician	Register‐based: Treatment, baseline symptoms, severity, age of onset Self‐report: CYBOCS‐based questions and survey questions	Moderate
Schwartz‐Lifshitz et al. (2024) Israel	44.4% female	10–21	18	Pre‐pandemic (unspecified) and during the pandemic (April 15–May 5, 2020; April 15–May 15, 2022)	Longitudinal	OCD	Clinical assessment	Register‐based: Medication use Clinician‐rated: CGI Self‐report: OCI and survey questions	Moderate
Tanir et al. (2020) Turkey	44.3% female	6–18	61	Pre‐pandemic (September 2019–March 2020) and during the pandemic (March 11–April 22, 2020)	Longitudinal	OCD	Clinical assessment	Clinician‐rated: CGI and CYBOCS Self/parent‐report: Survey questions	Moderate
Akgül et al. (2022) Turkey	94.7% female	12–18	38	May 13–June 11, 2020	Cross‐sectional	ED	Register‐based	Self‐report: EDE‐Q, BDI, SAI, MOCI and survey questions	Moderate
Torun et al. (2024) Turkey	94.6% female	12–18	37	Pre‐pandemic (at the time of diagnosis) and during the pandemic: May–June 2020 (Akgül et al., 2021); May–June 2021 (current study)	Longitudinal	ED	Register‐based	Register‐based: Medication use, BMI, target weight Self‐report: EDE‐Q, BDI, SAI, MOCI and survey questions	Moderate
Kuz et al. (2023) Turkey	69.8% female	12–18	63	During the pandemic (unspecified)	Cross‐sectional	Anxiety disorders	Register‐based	Parent‐report: SCARED Self‐report: CRIES, DERS‐SF	High
Sadeghi et al. (2022) USA	70.8% female (diagnosed) 58.6% female (without diagnoses)	11–17	96 (diagnosed) 70 (without diagnoses)	Pre‐pandemic (March 11, 2019–March 10, 2020) and during the pandemic (March 11, 2020–March 11, 2021)	Longitudinal	MDD	Standardized instrument (K‐SADS) administered by a clinician	Parent‐report or self‐report: SMFQ, SCARED Self‐report: CRISIS	Moderate
De Nardi et al. (2021) Italy	51.7% female (diagnosed) 45.6% female (without diagnoses)	13–18	58 (diagnosed) 57 (without diagnoses)	April 27–May 3, 2020	Cross‐sectional	SSD	Clinical assessment	Self‐report: MASC‐2‐SR, CDI‐2‐SF	High
Studies focusing on multiple types of pre‐existing conditions									
Bilu et al. (2023) Israel	39.5% female	12–17	∼42,204 (ND/diagnosed) ∼844,629 (NT/without diagnoses)	Pre‐pandemic (November 2016–February 2020) and during the pandemic (March 2020–October 2021)	Longitudinal	Neurodivergences (ADHD); a range of psychiatric disorders, including depression, anxiety, ED, OCD and stress‐related disorders	Register‐based	Register‐based	Low
Bliddal et al. (2023) Denmark	∼54% female	5–24	108,840 (total sample) Number of ND/diagnosed —N/A	Pre‐pandemic (January 1, 2017–February 29, 2020) and during the pandemic (March 1, 2020–June 30, 2022)	Repeated cross‐sectional	Neurodivergences (autism); a range of psychiatric disorders, including psychotic, mood, anxiety and hyperkinetic disorders	Register‐based	Register‐based	Moderate
Burrell et al. (2023) USA	54.3% female	12–17	5420	Pre‐pandemic (March 1, 2019–February 28, 2020) and during the pandemic (March 1, 2020–February 28, 2021)	Longitudinal	Neurodivergences (ADHD); a range of psychiatric disorders, including anxiety, mood disorders, dual diagnoses of anxiety and mood disorders, psychosis and multiple conditions	Register‐based	Register‐based	Moderate
Conti et al. (2020) Italy	13.1% female (preschool group) 20% female (school group)	1.5–5 6–18	61 (preschool group) 80 (school group)	Pre‐pandemic (September 2019–February 2020) and during the pandemic (April 20–May 4, 2020)	Longitudinal	Neurodivergences (e.g., autism, ADHD, ID); emotional and behavioral disorders (OCD, anxiety, mood and feeding disorders)	Multispecialist clinical assessment and standardized instruments (e.g., K‐SADS)	Parent‐report: CBCL	High
Erez et al. (2022) Israel	∼54.6% female	≤18	1309 → 1112 (total) 425 → 484 (those with pre‐existing conditions)	Pre‐pandemic (March–December 2019) and during the pandemic (March–December 2020)	Repeated cross‐sectional	Neurodivergences (ADHD); a range of psychiatric disorders, including anxiety, mood, conduct and stress‐related disorders	Register‐based	Register‐based	Moderate
Gilsbach et al. (2021) Germany	55.5% female (ND/diagnosed) 33.3% female (NT/without diagnoses)	6–18	144 (ND/diagnosed) 48 (NT/without diagnoses)	Spring 2020	Cross‐sectional	Neurodivergences (ADHD); a range of psychiatric disorders, including depression, anxiety disorders, ED, OCD and PTSD	Register‐based	Parent‐report: PROPS Self‐report: CROPS Parent‐report and self‐report: Survey questions	Moderate
Haller et al. (2024) USA	∼40.7% female	8–18	47 (ND/diagnosed) 34 (NT/without diagnoses)	Pre‐pandemic (*M* = 7.2 months before the pandemic) and during the pandemic (*M* = 6.5 months after the start of the pandemic)	Longitudinal	Neurodivergences (ADHD); a range of psychiatric disorders, including DMDD, ODD and anxiety disorders	Standardized instrument (K‐SADS) administered by a clinician	Parent‐report and self‐report: SMFQ, SCARED Parent‐report: CRISIS	Moderate
Khan et al. (2022) Qatar	44.4% female	14–18	63	During the pandemic (unspecified)	Cross‐sectional	Neurodivergences; a range of psychiatric disorders, including OCD, mood, anxiety and conduct disorders	Clinical assessment	Self‐report: OCI and COVID‐19 inventory (adapted from Swine Flu inventory)	Moderate
Lewis et al. (2023) USA	∼44.9% female	15–17	117 (with ADHD) 38 (with an internalizing disorder) 81 (without ADHD or internalizing disorder)	During the pandemic (spring 2020, summer 2020, fall 2020, spring 2021)	Longitudinal	Neurodivergences (ADHD) and internalizing disorders (anxiety and depressive disorders)	Standardized instrument (ChIPS) administered under clinician's supervision	Self‐report: CATS	Moderate
Lund et al. (2025) Norway	48.6% female	6–19	∼898,805	Pre‐pandemic (January 2017–February 2020) and during the pandemic (March 2020–December 2021)	Longitudinal	Neurodivergences (ADHD); a range of psychiatric disorders, including anxiety and depression	Register‐based	Register‐based	Moderate
Operto, Coppola, et al. (2022) Italy	36.4% female	2–18	217	Pre‐pandemic (September 2019–January 2020) and during the pandemic (March–May 2020)	Longitudinal	Neurodivergences (autism, ADHD, ID); a range of psychiatric disorders, including anxiety, mood and behavioral disorders	Multispecialist clinical assessment and standardized instruments (e.g., ADOS‐2, K‐SADS)	Parent‐report: CBCL and survey questions	Moderate
Operto, Scaffidi Abbate, et al. (2022) Italy	39.5% female	12–18	86	Pre‐pandemic (September 2019–January 2020) and during the pandemic (March–May 2020)	Longitudinal	Neurodivergences (autism, ADHD, ID); a range of psychiatric disorders, including anxiety, mood and behavioral disorders	Multispecialist clinical assessment and standardized instruments	Parent‐report: CBCL	Moderate
Parlatini et al. (2024) England	44.8% female	5–17	2503	June 15–September 2, 2020	Cross‐sectional	Neurodivergences (autism, ADHD, ID) and emotional disorders (depression, anxiety disorders, PTSD and OCD)	Register‐based	Parent‐report: Survey questions	Moderate
Payne et al. (2023) Australia	56.4% female 0.9% non‐binary	6–18	110	July 22–November 30, 2020	Cross‐sectional	Neurodivergences (ADHD); a range of psychiatric disorders, including mood, anxiety, ED, OCD and stress‐related disorders	Register‐based	Parent‐report: CRISIS	Moderate
Şentürk Pilan et al. (2021) Turkey	32% female	7–12	50	Pre‐pandemic (unspecified) and during the pandemic (2020 lockdown, further unspecified)	Longitudinal	Neurodivergences (ADHD, ID); a range of psychiatric disorders, including anxiety disorders, psychosis and PTSD	Register‐based	Clinician‐rated: CGI Self‐report: Survey questions	Moderate
Şentürk Pilan et al. (2023) Turkey	61% female	12–18	59	Pre‐pandemic (unspecified) and during the pandemic (June–July 2020; December 2020–January 2021)	Longitudinal	Neurodivergences (autism, ADHD); a range of psychiatric disorders, including MDD, anxiety disorders, conduct disorders and OCD	Register‐based	Clinician‐rated: CGI Self‐report: Survey questions	Moderate
Shankar et al. (2022) USA	∼58.5% female	5–17	5278 → 2849 (total) 1211 → 719 (those with pre‐existing conditions)	Pre‐pandemic (March 2018–February 2020) and during the pandemic (March 2020–February 2021)	Repeated cross‐sectional	A range of psychiatric disorders (unspecified)	Register‐based	Register‐based	Moderate
Stevanovic et al. (2022) Georgia, Lithuania, Romania, Serbia and Turkey	44.4% female	5–18	421	May–August 2020	Cross‐sectional	Neurodivergences (autism, ADHD); a range of psychiatric disorders, including anxiety, mood, OCD, psychotic and conduct disorders	Clinical assessment	Self/parent‐report: Survey questions	Moderate
Zijlmans et al. (2021) Netherlands	43.8% female (ND/diagnosed) 52.6% (NT/without diagnoses)	8–18	249 (ND/diagnosed) 844 (NT/without diagnoses)	April 30–May 12, 2020	Cross‐sectional	Neurodivergences (autism, ADHD); a range of psychiatric disorders (e.g., depression)	Clinical assessment	Self‐report: PROMIS measures Parent‐report and self‐report: Survey questions	Moderate

*Note*: ABAS‐II, adaptive behavior assessment system‐second edition; ABC‐J, aberrant behavior checklist; ADHD, attention‐deficit/hyperactivity disorder; ADI‐R, autism diagnostic interview‐revised; ADOS‐2, autism diagnostic observation schedule‐second edition; ARI, affective reactivity index; AuBC, autism behavior checklist; BDI, Beck's depression inventory; BMI, body mass index; BPM, brief problem monitor; CARS, childhood autism rating scale; CATS, child and adolescent trauma screen; CBCL, child behavior checklist; CDI‐2‐SF, children's depression inventory short form; CGI, clinical global impression; ChIPS, children's interview for psychiatric syndromes; CPRS, Conner's parent rating scale; CRIES, children's revised impact of event scale; CRISIS, CoRonavIruS health impact survey; CRISIS_AFAR, CoRonavIruS health and impact survey adapted for autism and related neurodevelopmental conditions; CROPS, child report of post‐traumatic symptoms; CYBOCS, children's Yale‐Brown obsessive‐compulsive scale; DERS‐SF, difficulties in emotion regulation scale‐short form; DMDD, disruptive mood dysregulation disorder; ED, eating disorder; EDE‐Q, eating disorders examination questionnaire; HoNOSCA, health of the nation outcome scale for children and adolescents; ID, intellectual disability; K‐SADS, kiddie schedule for affective disorders and schizophrenia for school‐age children; MASC‐2‐SR, multidimensional anxiety scale for children 2nd edition‐self‐report; MDD, major depressive disorder; MOCI, Maudsley obsessive‐compulsive inventory; N/A, not available; ND, neurodivergent; NT, neurotypical; OCD, obsessive‐compulsive disorder; OCI, obsessive‐compulsive inventory; ODD, oppositional defiant disorder; PROMIS, patient‐reported outcomes measurement information system; PROPS, parent report of post‐traumatic symptoms; PTSD, post‐traumatic stress disorder; RBS‐R, repetitive behavior scale‐revised; RCADS, revised child anxiety and depression scale; SAI, state anxiety inventory for children; SCARED, screen for child anxiety related disorders; SCQ, social communication questionnaire; SDQ, strengths and difficulties questionnaire; SMFQ, short mood and feelings questionnaire; SNAP‐IV, Swanson, Nolan and Pelham rating scale; SRS‐2, social responsiveness scale‐2; SSD, somatic symptom disorder; SWAN, strengths and weaknesses of attention‐deficit/hyperactivity disorder symptoms and normal behavior scale; T‐DSM‐IV‐S, Turgay DSM‐IV disruptive behavior disorders rating scale; TIDES, the irritability and dysregulation of emotion scale; TOCS, Toronto obsessive‐compulsive scale; USA, United States of America; VABS‐II, Vineland adaptive behavior scales‐second edition; VADRS, Vanderbilt ADHD diagnostic rating scale.

^a^ The use of “∼” in the Gender ratio column signifies that the listed percentages represent approximate proportions of female participants based on the total sample; these studies did not provide full information about the gender of youth with pre‐existing conditions.

^b^ The overall risk of bias (RoB) was determined using five criteria, including recruitment/sampling strategy, sample size adequacy, outcome assessment, attrition/response rate and control for confounding. Lower ratings signified a lower RoB. Further details on the assessment of the RoB are available in Supporting Information S1.

**FIGURE 2:**
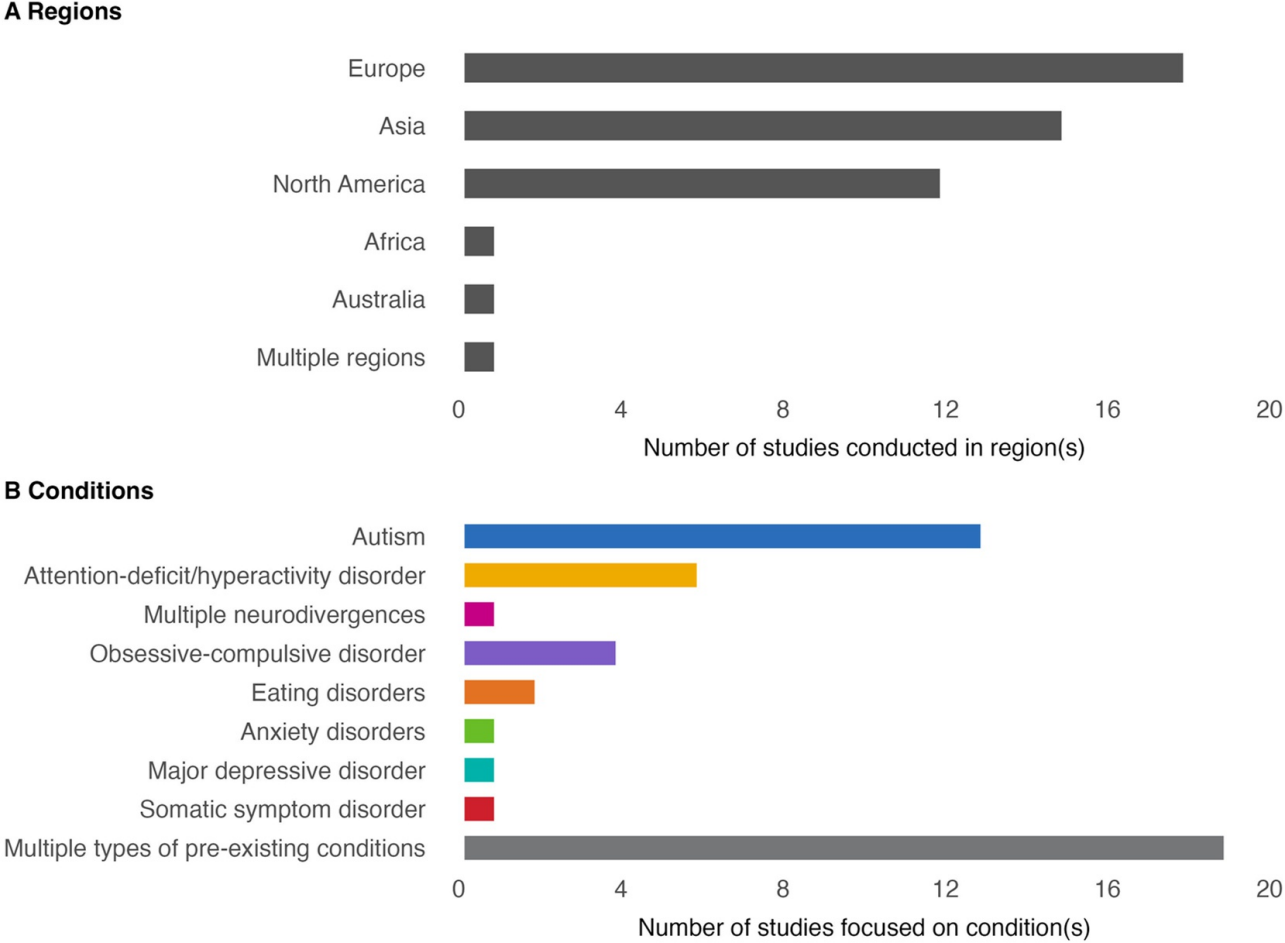
Regions and conditions covered by included studies. *N* = 48. (A) A further breakdown of studies by country is available in Supporting Information S1 (Table S3).

**FIGURE 3:**
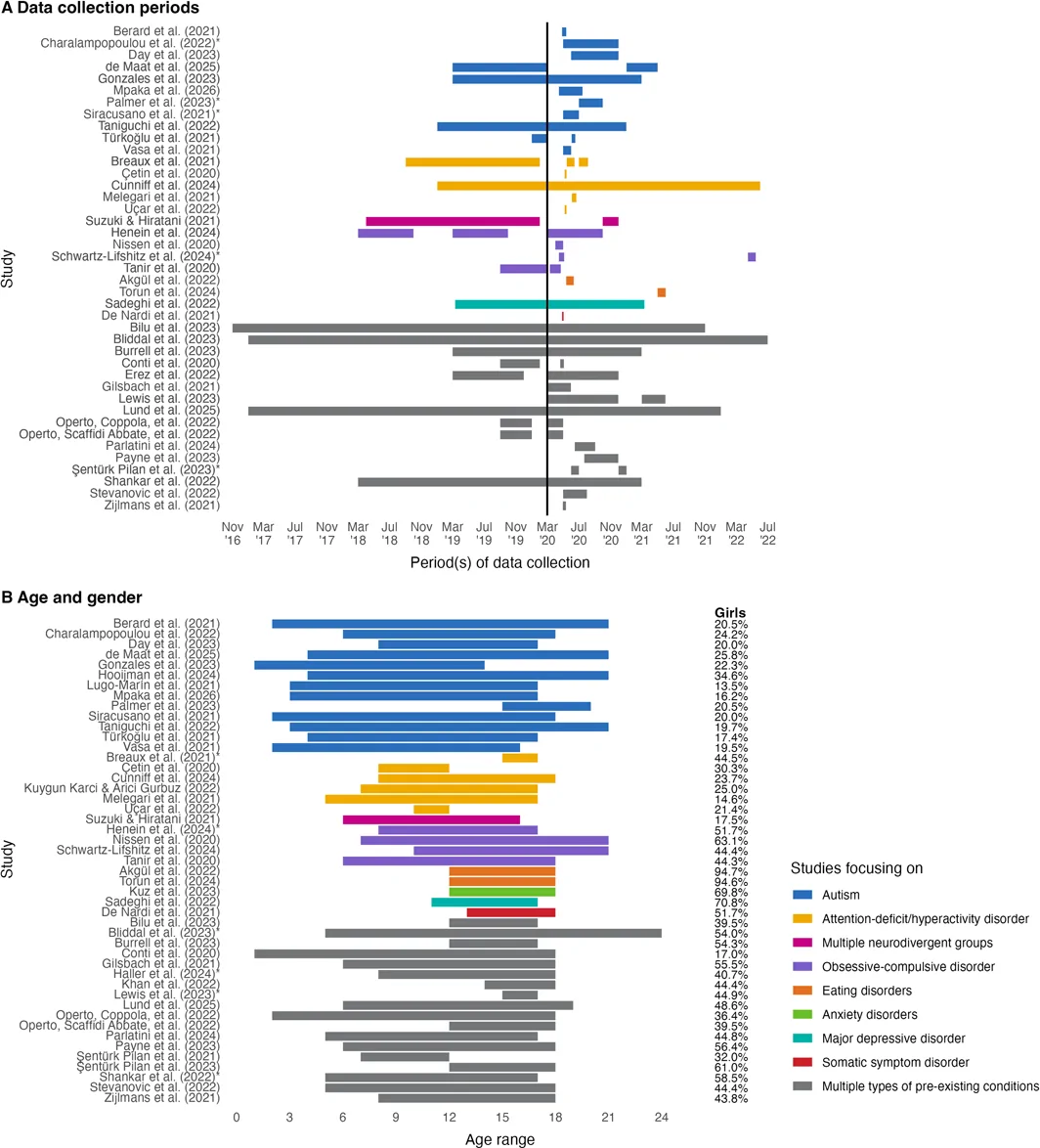
Data collection periods, age ranges and gender proportions across included studies. (A) *N* = 41. Out of 48 included studies, seven (Haller et al., 2024; Hooijman et al., 2024; Khan et al., 2022; Kuygun Karci & Arici Gurbuz, 2022; Kuz et al., 2023; Lugo‐Marín et al., 2021; Şentürk Pilan et al., 2021) were not added to this graph, because they did not provide information about the exact months of data collection (referring more generally to the pandemic and/or lockdowns instead). Studies with an asterisk (*) measured youth outcomes both before and during COVID‐19 but did not specify the exact months of pre‐pandemic data collection. The solid vertical line represents the official start of the pandemic, March 2020. (B) *N* = 47. One study (Erez et al., 2022) was not included in this graph, because it did not provide the exact age range. In studies with an asterisk (*), shown percentages represent approximate proportions of female participants based on the *total* sample; these studies did not provide full information about the gender of youth with pre‐existing conditions. Out of all included studies, only one study (Payne et al., 2023) reported on gender‐diverse youth, and they represented only a very small proportion of the sample (0.9%). Other studies only used and/or reported the binary categories (i.e., girls, boys).

### Studies focusing on a single neurodivergent group

#### Autistic youth

Considerable variation was found in the mental health of autistic youth during the pandemic, both within (Day et al., 2023; de Maat et al., 2025) and across studies. While some longitudinal studies reported an increase in internalizing and/or externalizing symptoms (Charalampopoulou et al., 2022; Türkoğlu et al., 2021), other studies found stability (de Maat et al., 2025; Lugo‐Marín et al., 2021; Siracusano et al., 2021) or improvements in these symptoms (Day et al., 2023). Most studies on autistic youth relied on parent‐report. Only one longitudinal study utilized both self‐ and parent‐report for the same measure (Day et al., 2023), yielding inconsistent findings across informants.

Looking at potential moderators, autistic youth were more likely to experience mental health symptoms during the pandemic if they had comorbid conditions (e.g., ADHD, ID), showed higher levels of irritability, had greater awareness of the pandemic, experienced greater pandemic‐related stress or lost access to academic and medical services (Charalampopoulou et al., 2022; Mpaka et al., 2026; Siracusano et al., 2021; Türkoğlu et al., 2021; Vasa et al., 2021). Conversely, continued access to interventions (e.g., behavioral or educational services) was associated with improvements in the communication and adaptive functioning of autistic youth during the pandemic (Berard et al., 2021; Siracusano et al., 2021). Higher pre‐pandemic symptoms were linked to either higher (Charalampopoulou et al., 2022; Hooijman et al., 2024; Palmer et al., 2023) or lower symptom levels (de Maat et al., 2025; Lugo‐Marín et al., 2021) during the pandemic.

Studies that examined healthcare utilization showed that autistic youth attended fewer hours of behavioral services during the first 3 months of the pandemic, but their use increased in the following 9 months (Gonzales et al., 2023). Autistic youth with a comorbid condition were more likely to return to the clinic during the pandemic (Taniguchi et al., 2022). Changes in pharmacological treatment (due to worsening of their state) occurred more often among autistic youth with a higher severity level than among those with a lower severity level (Lugo‐Marín et al., 2021).

Gender was not associated with changes in mental health symptoms or service utilization among autistic youth during the pandemic (Berard et al., 2021; Charalampopoulou et al., 2022; de Maat et al., 2025; Hooijman et al., 2024; Mpaka et al., 2026; Siracusano et al., 2021; Taniguchi et al., 2022; Vasa et al., 2021). Findings on the role of age of autistic youth during the pandemic were mixed. In some studies, younger age was a protective factor, linked to improved adaptive functioning and communication during the pandemic (Berard et al., 2021; Siracusano et al., 2021). Meanwhile, in other studies age did not predict the outcomes (Day et al., 2023; de Maat et al., 2025; Hooijman et al., 2024; Lugo‐Marín et al., 2021; Mpaka et al., 2026; Palmer et al., 2023; Vasa et al., 2021). Findings on the role of age in healthcare utilization were also inconsistent. Two studies reported greater use of healthcare services among older youth compared to their younger peers during the pandemic (Charalampopoulou et al., 2022; Taniguchi et al., 2022), while another study found the opposite pattern (Gonzales et al., 2023).

#### Attention‐deficit/hyperactivity disorder

Among youth with ADHD, there was also substantial variation in outcomes and moderating factors during the pandemic. For instance, ADHD diagnosed prior to the pandemic predicted increased externalizing symptoms, but not internalizing symptoms, during the pandemic (Breaux et al., 2021). Youth mental health during the pandemic was further moderated by individual factors and clinical characteristics. Specifically, youth with poorer emotion regulation showed higher inattention and hyperactivity/impulsivity (Breaux et al., 2021), whereas those with more severe pre‐pandemic internalizing and externalizing symptoms showed improvements in irritability, restlessness and opposition during the pandemic (Melegari et al., 2021). Contextual stressors and supports also played a role, with greater pandemic‐related stress linked to elevated oppositional defiant symptoms, irritability, depression and anxiety (Uçar et al., 2022; Çetin et al., 2020), while pre‐pandemic participation in organized sports was associated with fewer externalizing symptoms (Kuygun Karci & Arici Gurbuz, 2022). Most studies used parent‐report measures, and some studies also used self‐reports. There were no clear differences between informants.

During the pandemic, there was a decrease in the number of youth refilling their ADHD medication and attending regular clinic appointments compared to the levels before the pandemic (Cunniff et al., 2024; Kuygun Karci & Arici Gurbuz, 2022). Generally, the medication use did not return to pre‐pandemic levels when the restrictions were eased (Cunniff et al., 2024). Medication use was also higher among those who attended both online and in‐person appointments (Cunniff et al., 2024) or had a comorbid condition (Kuygun Karci & Arici Gurbuz, 2022).

In longitudinal studies with larger sample sizes (*n* = 118–992), boys were more likely to experience an increase in hyperactivity/impulsivity and take ADHD medication more regularly during the pandemic (Breaux et al., 2021; Cunniff et al., 2024) compared to girls. No gender differences were seen in oppositionality or inattention (Breaux et al., 2021). Younger youth with ADHD were more likely to have an increase in mood‐related problems, to refill their ADHD medication and attend clinic appointments, compared to older youth with ADHD (Cunniff et al., 2024; Melegari et al., 2021). In a cross‐sectional study with a smaller sample size (*n* = 42), gender and age did not predict the pandemic‐related stress (Uçar et al., 2022).

### Neurodivergent group comparison

One study specifically focused on mental health of multiple neurodivergent groups during the pandemic (Suzuki & Hiratani, 2021). However, other studies also provided relevant information for this section, despite their main focus on autistic youth (Mpaka et al., 2026; Siracusano et al., 2021) or multiple types of pre‐existing conditions (Operto, Scaffidi Abbate, et al., 2022; Parlatini et al., 2024). All these studies used parent‐report measures. None of the included studies compared healthcare utilization across neurodivergent groups.

Two out of three studies which compared mental health of autistic youth and youth with ADHD during the pandemic found greater vulnerability among autistic youth. In a longitudinal study, autistic youth had an increase in various domains of both internalizing and externalizing symptoms, while youth with ADHD only had an increase in somatic complaints and no changes in other domains (Operto, Scaffidi Abbate, et al., 2022). Similarly, in a cross‐sectional study, autistic youth experienced a greater worsening of internalizing symptoms and a higher level of pandemic‐related worries compared to youth with ADHD (Parlatini et al., 2024). However, in that study, no differences were observed in externalizing symptoms. In contrast, another longitudinal study (Suzuki & Hiratani, 2021) found no differences in mental health outcomes between these groups.

Across studies, comorbid conditions appeared to shape mental health outcomes during the pandemic, but patterns differed by comorbidity type. Autistic youth with co‐occurring ADHD showed greater externalizing and internalizing symptoms during the pandemic, compared to youth belonging to a single neurodivergent group (Parlatini et al., 2024). Findings on the mental health of autistic youth with ID during the pandemic were mixed, with opposite trends across studies (Mpaka et al., 2026; Parlatini et al., 2024; Siracusano et al., 2021). Notably, youth with comorbid ADHD and ID showed lower internalizing symptoms during the pandemic, compared to youth with ADHD alone (Parlatini et al., 2024), suggesting potential differences in vulnerability across comorbidity profiles. There was no information on how gender and age could further moderate differences across neurodivergent groups.

### Studies focusing on a single pre‐existing psychiatric disorder

#### Obsessive‐compulsive disorder

Across four studies, 21%–54% of youth experienced a worsening of their obsessive‐compulsive disorder (OCD) symptoms during the pandemic, while 18%–46% had improvements (Henein et al., 2024; Nissen et al., 2020; Schwartz‐Lifshitz et al., 2024; Tanir et al., 2020). In this group, most studies used self‐report and/or clinicians' ratings, with no clear differences between informants (Schwartz‐Lifshitz et al., 2024).

Comorbid conditions were not associated with increased OCD symptoms during the pandemic in either longitudinal (Schwartz‐Lifshitz et al., 2024) or cross‐sectional studies (Nissen et al., 2020). However, earlier age of onset, baseline sexual/aggressive OCD symptoms (Nissen et al., 2020), longer duration of OCD diagnosis and pandemic‐related worries (Tanir et al., 2020) predicted greater worsening of OCD symptoms. Findings on pandemic‐related changes in healthcare utilization among youth with pre‐existing OCD were limited, with one study reporting that fewer youth received medicated treatment during the pandemic (Tanir et al., 2020). Another study showed that receiving psychiatric therapy or taking serotonin reuptake inhibitors (SRIs) during the pandemic were not statistically significant predictors of OCD symptoms during the same period (Nissen et al., 2020).

Gender differences were analyzed in two studies, yielding inconsistent findings. A cross‐sectional study found that gender was not associated with OCD severity during the pandemic (Nissen et al., 2020). A longitudinal study found that girls with pre‐existing OCD were more likely to experience a worsening of OCD symptoms and take SRIs than boys during the pandemic (Schwartz‐Lifshitz et al., 2024). Findings on the role of age were limited, with only a cross‐sectional study showing that younger age was associated with a greater symptom worsening during the pandemic (Nissen et al., 2020).

#### Eating disorders

Evidence on youth with pre‐existing eating disorders (EDs) during the pandemic was limited to two studies, both focused on self‐report. In the first study, conducted at the end of Spring 2020, most youth (78.9%) did not report worsening of ED symptoms during the pandemic, although there was an increase in clinical depression compared to pre‐pandemic rates (Akgül et al., 2022). The second study using the same sample was conducted 1 year later (Torun et al., 2024). Compared to the first pandemic measurement, youth with pre‐existing EDs experienced an increase in anxiety but no change in depressive, ED and OCD symptoms during the second pandemic measurement. Depressive symptoms were identified as the strongest predictor of ED behaviors during the early and later stages of the pandemic, followed by anxiety at the later stage.

Findings from these two studies suggest variability in healthcare access among youth with EDs across the pandemic periods. In one study conducted during the first year of COVID‐19, about one in every four youth with EDs (23.7%) felt that the pandemic often negatively affected their access to healthcare services (Akgül et al., 2022). In the second year of the pandemic, the proportion of participants who often had difficulty in accessing ED healthcare substantially decreased, reaching 2.7% (Torun et al., 2024). The percentages of youth using psychotropic medication stayed stable across two pandemic stages (Torun et al., 2024).

Given that over 90% of participants were female, the studies on youth with EDs were unable to examine gender differences in mental health during the pandemic. Among youth with EDs, age was not correlated with anxiety, depression, OCD or ED symptoms during the pandemic (Torun et al., 2024).

#### Anxiety disorders, major depressive disorder and somatic symptom disorder

Evidence for youth with pre‐existing anxiety disorders (ADs), major depressive disorder (MDD) and somatic symptom disorder (SSD) was limited to one study per condition. In a cross‐sectional study of youth with pre‐existing ADs, self‐reported pandemic‐related stress was associated with greater self‐reported emotion regulation problems and parent‐reported anxiety (Kuz et al., 2023). In a longitudinal study, youth with pre‐existing MDD showed a modest reduction in internalizing symptoms during the pandemic, similar across the informants (Sadeghi et al., 2022). However, their symptom levels during the pandemic were still higher than those of controls without a psychiatric disorder. In contrast, another cross‐sectional study showed that youth with pre‐existing SSD had lower self‐reported internalizing symptoms compared to controls during the pandemic (De Nardi et al., 2021). There were no findings on pandemic‐related changes in healthcare utilization among youth with these pre‐existing disorders, except for the decrease in psychological treatment among youth with SSD (De Nardi et al., 2021).

Patterns of gender differences were similar across ADs and MDD. Compared to girls, boys were less likely to experience severe psychological stress during the pandemic (Kuz et al., 2023) and more likely to experience a decrease in self‐reported depressive symptoms (Sadeghi et al., 2022). While no age differences were found for youth with ADs (Kuz et al., 2023), older age was a protective factor against depressive symptoms among youth with MDD (Sadeghi et al., 2022). The study on SSD did not present any findings on gender or age differences (De Nardi et al., 2021).

### Studies focusing on multiple pre‐existing conditions

Substantial variation by informant was observed in studies including multiple pre‐existing conditions. Across studies using parent report, approximately 44%–52% of parents reported a worsening in their child's symptoms during the pandemic (Operto, Coppola, et al., 2022; Payne et al., 2023; Stevanovic et al., 2022), with increases in both internalizing and externalizing symptoms across a range of conditions, including autism, anxiety, mood and behavioral disorders (Operto, Coppola, et al., 2022; Operto, Scaffidi Abbate, et al., 2022). Across studies using self‐report, rates of perceived symptom deterioration were lower, typically ranging from 26% to 44% (Stevanovic et al., 2022; Şentürk Pilan et al., 2021). Interestingly, in a longitudinal study including both types of informants, youth with pre‐existing conditions reported an increase in internalizing symptoms during the pandemic that was not reported by their parents (Haller et al., 2024). Findings based on clinician assessments were mixed. One study indicated an increase in the severity of cases (Şentürk Pilan et al., 2021), whereas another reported stable or improved clinician‐rated functioning during the pandemic (Şentürk Pilan et al., 2023).

Across studies focused on mental health symptoms, some studies presented their findings by condition, enabling cross‐condition comparison. In two studies including school‐aged children and adolescents, neurodivergent youth were found to experience greater mental health symptom deterioration during the pandemic compared to youth with pre‐existing psychiatric disorders, including OCD, depressive disorders and ADs (Conti et al., 2020; Parlatini et al., 2024). However, neurodivergent youth were not always more vulnerable than other groups. For example, youth with ADHD experienced less COVID‐related distress (Gilsbach et al., 2021) and psychological trauma symptoms (e.g., reexperiencing, arousal; Lewis et al., 2023) than youth with depressive disorders and ADs during the pandemic. Another cross‐diagnostic study compared youth with OCD to youth with mood disorders or ADs and, interestingly, found that the latter group had greater OCD symptoms during the pandemic (Khan et al., 2022).

Considering healthcare utilization, a decline in psychiatric service use was reported for youth with ADHD, psychosis or multiple conditions during the pandemic, whereas findings for anxiety and mood disorders were mixed (Burrell et al., 2023; Lund et al., 2025). During the pandemic, youth who experienced a psychiatric emergency were more likely to have pre‐existing conditions, compared to pre‐pandemic times (Erez et al., 2022; Shankar et al., 2022). Overall, about a third of parents reported declines in quality and quantity of youth mental health services during the pandemic (Parlatini et al., 2024; Payne et al., 2023; Stevanovic et al., 2022). Service dissatisfaction was higher in youth with multiple pre‐existing conditions and was linked to greater internalizing and externalizing symptoms during the pandemic (Parlatini et al., 2024; Stevanovic et al., 2022; Şentürk Pilan et al., 2023). In contrast, parent‐reported weekly hours of pre‐pandemic treatment were not associated with the symptom change during the pandemic (Conti et al., 2020).

Regarding psychotropic medication, studies reported an increase in the use of antidepressants, hypnotics and sedatives among youth with pre‐existing conditions, but no change in antipsychotics and anxiolytics during the pandemic (Bilu et al., 2023; Bliddal et al., 2023). Findings on psychostimulants varied in their direction of change across studies (Bilu et al., 2023; Bliddal et al., 2023).

Gender‐related findings were mixed. Across several cross‐sectional studies, girls were more likely to have internalizing symptoms and COVID‐related distress than boys during the pandemic (Gilsbach et al., 2021; Parlatini et al., 2024; Stevanovic et al., 2022; Zijlmans et al., 2021). In a longitudinal study, girls were more likely to exhibit psychological trauma symptoms than boys throughout the pandemic (Lewis et al., 2023). In other studies, gender was not associated with increased mental health symptoms during the pandemic (Khan et al., 2022; Operto, Scaffidi Abbate, et al., 2022; Payne et al., 2023). In terms of psychiatric healthcare, girls were more likely to have an increase in psychiatric service use than boys during the pandemic (Burrell et al., 2023; Lund et al., 2025), but there were no gender differences in the medication use (Bilu et al., 2023).

The association between age and mental health varied depending on the child's education stage (i.e., pre‐school, school) and outcome type (e.g., internalizing symptoms, externalizing symptoms). Overall, increases in internalizing and externalizing symptoms during the pandemic were observed in both preschoolers and school‐aged youth, but effect sizes were typically larger at the latter education stage, except for aggression (Operto, Coppola, et al., 2022). Among preschoolers, older age was linked to a greater worsening of total mental health problems and ADHD symptoms during the pandemic (Conti et al., 2020). For school‐aged youth, older age was associated with greater symptom worsening and internalizing symptoms (Parlatini et al., 2024; Payne et al., 2023) but lower externalizing symptoms (Parlatini et al., 2024; Zijlmans et al., 2021) during the pandemic. In other studies, age showed no association with the outcomes among school‐aged youth (Gilsbach et al., 2021; Haller et al., 2024; Khan et al., 2022; Operto, Scaffidi Abbate, et al., 2022). Findings on the role of age in service use were only available for adolescents, with younger adolescents (12–13.9 years) showing a larger relative increase in mental health service use, compared to older adolescents aged 14–15.9 years, during the pandemic (Burrell et al., 2023). Regarding psychotropic medication use, adolescents aged 12–17 years had more prominent relative increases in prescriptions during the pandemic, compared to children (5–11 years) and older youth (18–24 years; Bliddal et al., 2023). In a separate study focused on adolescents aged 12–17 years, anxiolytic use showed the greatest increase among 12–13‐year‐olds, whereas antidepressant use rose most prominently among those aged 14–15 years (Bilu et al., 2023).

### Youth with pre‐existing conditions compared to their peers without such conditions

Across *all* included studies, findings were inconsistent when mental health symptoms were compared between youth with pre‐existing conditions and neurotypical controls without prior psychiatric disorders. Six studies reported that youth with pre‐existing conditions tended to have greater internalizing and/or externalizing symptoms and COVID‐related distress than controls during the pandemic (Breaux et al., 2021; de Maat et al., 2025; Gilsbach et al., 2021; Lewis et al., 2023; Sadeghi et al., 2022; Zijlmans et al., 2021). However, some studies found no differences in the level of symptoms during the pandemic (Day et al., 2023). Similarly, while one study showed that youth with pre‐existing conditions were more likely to experience an increase in symptoms during the pandemic than controls (Breaux et al., 2021), another study found no difference in the mean change between the groups (de Maat et al., 2025). Furthermore, some studies found more symptom exacerbation (Haller et al., 2024; Sadeghi et al., 2022) and greater increases in medication use (Bilu et al., 2023) among controls without pre‐existing conditions. No findings were available on differences in mental health service use.

### Risk of bias in included studies

Based on the five indicators in the RoB assessment, 40 studies (83.3%) were rated as having a moderate overall RoB, seven studies (14.6%) were rated with high RoB, while one study (2.1%) met the threshold for low RoB (see Table 2 and Supporting Information S1: Table S4 for further details).

RoB concerns were most pronounced in ADs (Kuz et al., 2023) and SSD (De Nardi et al., 2021) categories. The only available studies for these categories received a high overall RoB rating due to concurrent high risks on two or three assessment criteria. Among studies on youth with ADHD, 2 (Kuygun Karci & Arici Gurbuz, 2022; Çetin et al., 2020) out of 6 available studies (33.3%) were rated as having a high overall RoB, following high‐risk ratings on three indicators. The proportion of high RoB studies was lower among studies on autistic youth. In this category, 2 (Lugo‐Marín et al., 2021; Türkoğlu et al., 2021) out of 13 studies (15.4%) were rated as high RoB due to high risk on two assessment criteria. Among studies examining multiple conditions, only 1 (Conti et al., 2020) out of 19 studies (5.3%) was rated with high overall RoB. This was also the only category to include a study rated with a low overall RoB (Bilu et al., 2023). No studies on OCD, ED, MDD and multiple neurodivergent groups were rated as having a high overall RoB.

Two of the five assessment criteria, recruitment/sampling strategy and sample size adequacy, accounted for most high overall RoB ratings. Recruitment/sampling strategy was rated as high‐risk in all 7 studies with a high overall RoB, while sample size adequacy was rated as high risk in 6 of the 7 studies. The single available studies for ADs and SSD also had non‐representative, smaller samples. Consequently, their 100% high‐RoB rate likely reflects the broader design‐related pattern rather than condition‐specific methodological shortcomings and should not be interpreted as evidence that research on these conditions is inherently of lower quality.

## DISCUSSION

This systematic review provides an up‐to‐date overview of findings on mental health symptoms and psychiatric healthcare use among neurodivergent youth and youth with pre‐existing psychiatric disorders during the COVID‐19 pandemic. The review found no uniform pattern of mental health change among youth with pre‐existing conditions, with findings varying both across and within diagnostic groups. In addition, individual studies identified associations of outcomes with gender, age, comorbid conditions, emotion regulation, pre‐pandemic symptom severity, irritability, exposure to pandemic‐related stress and access to care. Together, these findings suggest that considering diagnostic status alongside individual, developmental, contextual and service‐related factors may provide a more complete understanding of clinical needs during large‐scale societal disruptions.

Studies comparing different diagnostic groups were few, and their findings lacked consistency. Beyond the diagnosis, the impact of pandemic‐related changes on youth mental health outcomes could have been modified by their characteristics and lived experiences. The mixed findings among autistic youth may potentially illustrate this interplay, with some individuals having increased mental health difficulties during the pandemic and others having improvements or stability. These findings are consistent with previously reported interindividual differences among neurodivergent youth (Cruz Puerto & Sandín Vázquez, 2024) and suggest that their responses to routine disruptions and experienced minority stress (Botha & Frost, 2020) could interact with societal instability in complex ways. For some youth, reduced social demands and increased flexibility in educational delivery may have buffered distress (Day et al., 2023), whereas for others, service disruptions and heightened stress were associated with greater difficulties (Parlatini et al., 2024). Notably, the variability of findings across studies could also be related to differences in country‐level policies (e.g., COVID‐related restrictions) but detailed examination of policy variation was beyond the scope of this review.

Findings on healthcare utilization suggested a decrease in mental health service use among some youth. However, the available evidence was limited and largely restricted to the early phases of the pandemic, with data extending only to 2022 (Burrell et al., 2023; Lund et al., 2025). As a result, longer‐term patterns in service use among youth with pre‐existing conditions remain unclear. Studies examining psychotropic medication use highlighted increased use of antidepressants, hypnotics and sedatives during the pandemic (Bilu et al., 2023; Bliddal et al., 2023). Combined with widespread reports of declines in the quality and quantity of available mental health services (Parlatini et al., 2024; Payne et al., 2023; Stevanovic et al., 2022), these findings raise the possibility that pharmacological treatment may have partially compensated for reduced access to in‐person or specialist care during the pandemic. This assumption is consistent with prior qualitative reports of barriers to mental health service access during this period (Markoulakis et al., 2022). However, direct evidence linking reduced access to increased prescribing within the same population is limited.

Consistent with prior systematic reviews (Ching et al., 2025; Madigan et al., 2023), several studies reported that girls with a pre‐existing condition experienced greater internalizing symptoms, distress and trauma‐related outcomes than boys during the pandemic. Fewer studies examined gender differences in symptom changes from before to during the pandemic. The available evidence showed that girls were more likely to have an increase in OCD symptoms (Schwartz‐Lifshitz et al., 2024), whereas boys were more likely to have increases in externalizing symptoms (Breaux et al., 2021) and decreases in internalizing symptoms (Sadeghi et al., 2022). Extending previous work, this review also indicated that girls were more likely to have an increase in mental health service use than boys during the pandemic (Burrell et al., 2023; Lund et al., 2025), suggesting not only heightened symptom burden but also differential patterns of help‐seeking or service engagement. However, evidence of gender differences was not consistent across conditions. In particular, studies did not identify gender differences in mental health outcomes of autistic youth during the pandemic. This absence of evidence should be interpreted cautiously, given persistent underrepresentation of girls in autism research and ongoing concerns that diagnostic criteria may insufficiently capture autistic presentations in women (Botha & Cage, 2022). Similarly, scarce and mixed evidence regarding gender differences among youth with ADHD mirrors broader gaps in the ADHD literature (Babinski, 2024) and highlights the need to systematically examine gender as a moderator of mental health trajectories and service use in neurodivergent populations.

Findings related to age differences were mixed and appeared to vary across developmental stages and mental health outcomes. Rather than indicating a single period of heightened vulnerability, these patterns suggest that younger and older youth may have faced distinct challenges during the pandemic, shaped by developmental needs, educational context and social demands. From a clinical and service‐planning perspective, these findings highlight the importance of developmentally tailored approaches to assessment and intervention, rather than assuming uniform risk across age groups.

In addition to pre‐pandemic condition, gender and age, outcomes also varied depending on pre‐pandemic symptom severity, comorbid conditions, COVID‐related stress, access to healthcare, irritability and emotion regulation skills. Furthermore, findings were influenced by the informants. Studies using both self‐ and parent‐report revealed meaningful discrepancies, with youth reporting changes in internalizing symptoms more frequently than their parents (Day et al., 2023; Haller et al., 2024). This pattern is consistent with established guidelines which emphasize the use of self‐report for internalizing symptoms (Aebi et al., 2017). Together, these findings underscore the need for personalized approaches to identifying clinical needs (Bozzola et al., 2022; Kroon et al., 2022), considering these factors, as well as for the use of multi‐informant data to more accurately capture mental health trajectories among youth with pre‐existing conditions during public health emergencies.

This systematic review has several limitations. First, a meta‐analysis was not conducted because the available studies were not sufficiently comparable across study designs, outcome measures, informants and types of comparison and several diagnostic categories were represented by very few studies. Consequently, we could not estimate pooled effect sizes, formally quantify between‐study heterogeneity or weight findings according to their statistical precision. The findings should therefore be interpreted as patterns across the available literature rather than as pooled estimates of pandemic‐related effects. Second, this review focused on youth with confirmed pre‐existing conditions. Therefore, our findings may not generalize to those with unconfirmed neurodivergences and/or mental health difficulties. Third, evidence was uneven across pre‐existing conditions, limiting the strength of condition‐specific conclusions. In addition, diagnostically distinct conditions were often grouped together (e.g., ADs), which may have obscured clinically meaningful differences between specific disorders (Morales et al., 2022). The inclusion of cross‐sectional studies also limited conclusions about changes over time; however, these studies provided complementary evidence in diagnostic groups where longitudinal data were sparse. Other methodological limitations across studies included uneven geographical distribution, lack of representation of gender‐diverse individuals, reliance on retrospective recall or perceived change, potential confounding by time and wide variation in sample sizes. The majority of studies had a moderate overall RoB. High overall RoB concentrated in studies using smaller, non‐representative samples. Thus, differences in RoB in this review appear to reflect, at least partly, the designs of the available studies. This is particularly important for ADs and SSD, for which only one study was available in each category. Therefore, conclusions for these groups, as well as for ADHD where one‐third of studies had high overall RoB, should be interpreted cautiously.

## CONCLUSIONS

Our findings indicate that mental health outcomes of youth with pre‐existing conditions during the COVID‐19 pandemic varied both within and across diagnostic groups. Outcomes were also associated with individual, developmental, contextual and service‐related factors. These included gender, age, comorbid conditions, emotion regulation, pre‐pandemic symptom severity, irritability, exposure to pandemic‐related stress and access to care. These findings suggest the importance of considering such factors alongside diagnosis when identifying clinical needs and planning support during future large‐scale societal disruptions. Given the predominantly moderate overall RoB of included studies, more methodologically robust research is needed to clarify mental health and healthcare‐use patterns during major public health emergencies.

## AUTHOR CONTRIBUTIONS

**Ulyana Sirotina**: Conceptualization; methodology; formal analysis; visualization; writing—original draft; writing—review and editing; project administration; investigation. **Bryndis Lara Bjarnadottir**: Methodology; investigation; visualization; formal analysis; writing—review and editing. **Agnieszka Butwicka**: Conceptualization; methodology; funding acquisition; writing—review and editing. **Eivind Ystrom**: Conceptualization; methodology; funding acquisition; writing—review and editing. **Jaana Suvisaari**: Conceptualization; methodology; funding acquisition; writing—review and editing. **Olli Kiviruusu**: Conceptualization; methodology; funding acquisition; writing—review and editing. **Audun Valborgarson**: Conceptualization; methodology; writing—review and editing. **Heiddis Bjork Valdimarsdottir**: Conceptualization; methodology; writing—review and editing. **Helga Ask**: Conceptualization; methodology; writing—review and editing; funding acquisition. **Unnur Valdimarsdottir**: Conceptualization; methodology; funding acquisition; writing—review and editing. **Thorhildur Halldorsdottir**: Conceptualization; methodology; investigation; funding acquisition; writing—review and editing; supervision.

## CONFLICT OF INTEREST STATEMENT

E.Y. is a Joint Editor for *JCPP Advances*. The remaining authors have declared that they have no competing or potential conflicts of interest. See Acknowledgments for full disclosures.

## ETHICAL CONSIDERATIONS

Since the work presented in this manuscript is a systematic review, ethical approval and informed consent were not required.

## TRIAL REGISTRATION

This review was conducted in accordance with the PRISMA guidelines, and its protocol has been pre‐registered on PROSPERO (CRD420251005264) on 15/03/2025.

## Supporting information

Supporting Information S1

## Data Availability

Data sharing not applicable to this article as no datasets were generated or analyzed during the current study.
