# Supplementary material for: Mental health and healthcare use during COVID‐19 among neurodivergent youth and youth with pre‐existing psychiatric disorders: A systematic review
Source: JCPP Adv. 2026 Oct 8:e70176. Online ahead of print. doi: 10.1002/jcv2.70176 (PMC13650191; doi:10.1002/jcv2.70176)
Supplement: Supplementary file 1 [file JCV2-9999-e70176-s001.docx]

**Mental Health and Healthcare Use During COVID-19 among Neurodivergent Youth and Youth with Pre-Existing Psychiatric Disorders: A Systematic Review**

**Supporting Information**

**Appendix S1. Methods**

**Search strategy**

The search strategy was developed by this review’s authors. It was then reviewed by the university librarian, not otherwise associated with this project, who provided useful feedback.

As a result, Web of Science (WoS) was searched using the following query:

TI=(covid) AND TI=(child* OR adolesc* OR pediatric OR youth OR teen*) AND ALL=(mental health disorder OR psychiatric disorder OR neurodevelopmental disorder OR depr* OR anxi* OR ADHD OR autis* OR ASD OR "intellectual disability" OR dysthymi* OR phobi* OR "conduct disorder" OR "eating disorder" OR anorexi* OR bulimi* OR bing* OR "obsessive-compulsive disorder" OR OCD OR "oppositional defiant disorder") AND ALL=(diagnosis OR pre-existing)

The following limits were applied to the WoS query:

- Language: English
- Publication years: from 2020 to present

PubMed was searched using the following query:

(covid[Title]) AND (child*[Title] OR adolesc*[Title] OR pediatric[Title] OR youth[Title] OR teen*[Title]) AND (mental health disorder OR psychiatric disorder OR neurodevelopmental disorder OR depr* OR anxi* OR ADHD OR autis* OR ASD OR "intellectual disability" OR dysthymi* OR phobi* OR "conduct disorder" OR "eating disorder" OR anorexi* OR bulimi* OR bing* OR "obsessive-compulsive disorder" OR OCD OR "oppositional defiant disorder") AND (diagnosis OR pre-existing)

The following limits were applied to the PubMed query:

- Article language: English
- Publication date (custom range): from 2020 to present
- Species: Human
- Age: Child

*Note.* We did not *specifically* search for schizophrenia spectrum and other psychotic disorders, bipolar disorders and personality disorders, because these conditions are relatively rare in youth. However, if there were any findings available on these conditions, they were included in this review. On the other hand, we excluded studies which focused solely or primarily on learning disabilities, as these conditions mainly affect academic skills, rather than emotional and behavioral regulation, and are managed through educational interventions, rather than mental health services.

**Studies which were excluded from the review**

Based on the PRISMA flow diagram available in the manuscript, the main reason for excluding papers was the lack of an officially confirmed diagnosis (i.e., diagnosis confirmed by a clinician or through medical records). Some relevant papers, such as Kroon et al. (2022), Sicouri et al. (2023) and Cost et al. (2022), relied on parent-reported diagnoses and, as a result, could not be included in this review.

**Data extraction and synthesis**

Youth’ gender was recorded as reported by youth, their parents or clinicians. We used the terms girls and boys if these were the gender categories available in the study. Non-binary and transgender identities were also reported if they were available.

**Risk of bias in individual studies**

To create a list of risk of bias (RoB) indicators, we partly followed an approach outlined by Ching et al. (2025), adjusting it for the inclusion of cross-sectional studies. Thus, our list of RoB indicators was based on Newcastle-Ottawa Scale, National Institute of Health Quality Assessment Tool for Observational Cohort and Cross-Sectional Studies and Critical Appraisal Skills Programme Checklist for Cohort Studies. It consisted of the following questions:

1. Were sampling methods used to achieve a representative sample of the population being studied, e.g., use of quota sampling or probability sampling to reflect demographics of wider population? **(Sampling and recruitment)**

- **Low risk:** such sampling methods were used
- **Moderate risk:** such methods were not used, but the study demonstrated efforts to make the sample comparable to the studied population in terms of demographics
- **High risk:** efforts to address sampling bias were not described; the sampling bias is discussed in the limitations section

2. Was a sample size justification, power description, or variance and effect estimates provided? **(Sample size)**

- **Low risk:** the sample size is well described and justified; variance/effect estimates are provided
- **Moderate risk:** there is some information on these matters, but it is limited
- **High risk:** no information is provided on these matters; the sample size is very small

3. How were the outcomes measured? Was there an assessment/interview administered by a clinician and/or were clinical records used? If not and self-report/parental report questionnaires were used instead, were those standardized? **(Outcome assessment)**

- **Low risk:** an outcome assessment was done by a clinician or clinical records were used
- **Moderate risk:** standardized self-report/parent questionnaires were used; the clinician/clinical records were not involved in the outcome assessment
- **High risk:** only unstandardized measures were used

4. Was attrition/loss to follow-up after baseline 30% or less and were appropriate strategies used to address attrition, e.g., analysis of whether attrition occurred at random and if not, strategies for selective attrition, such as weighting? In case of cross-sectional studies, were the characteristics of participants who responded and did not respond comparable and/or the response rate was satisfactory (i.e., higher than 50%)? **(Attrition)**

- **Low risk:** the attrition/non-response rate was equal to or lower than the suggested estimate; appropriate strategies to address attrition/non-response were used, e.g., differences between groups (e.g., lost versus retained participants) were reported (unless the loss is very minimal)
- **Moderate risk:** the attrition/non-response rate was slightly higher than the suggested estimate; appropriate strategies to address attrition/non-response were not described, e.g., differences between groups were not reported
- **High risk:** the attrition/non-response rate was not reported, or it was considerably higher than the suggested estimate; appropriate strategies to address attrition/non-response were not described

5. Were confounding factors considered in the design and/or analysis, e.g., adjusted statistically? **(Confounding)**

- **Low risk:** factors important for the study were controlled for
- **Moderate risk:** the study controlled for some but not all important factors
- **High risk:** no factors were reported to be controlled for

The overall RoB was determined by giving ratings of “high”, “moderate”, “low” or “not applicable” for each indicator. Lower ratings signified a lower risk of bias. Each study received an overall RoB rating based on the listed indicators. A study was classified as *high* RoB if at least two indicators were rated high without any low ratings, and *low* RoB only if all indicators were rated low. All other cases were classified as *moderate* RoB.

**Appendix S2. Results**

**Table S1**

*Main Relevant Findings Across Included Studies*

| **Authors, country** | **Pre-existing condition** | **Main relevant findings** |
| --- | --- | --- |
| Studies focusing on a single ND group | | |
| Berard et al. (2021)  France | Autism | More than a third of parents reported that their children had a deterioration in challenging and stereotyped behaviors during the pandemic. 28.8% of parents also noticed improvements in communication with their children. Interventions were interrupted for 24.2% of youth.  Younger age and better daily living skills were protective factors against increased challenging behaviors. Younger age and continuity of interventions predicted improved communication.  Gender did not predict changes in any of these behaviors. |
| Charalam- popoulou et al. (2022)  Canada | Autism | Based on parents’ ratings, 61.3% of youth experienced a deterioration in all measures of mental health, including internalizing symptoms (mood, anxiety, OCD symptoms) and externalizing symptoms (irritability, ADHD symptoms).  Higher pre-COVID internalizing symptoms and COVID-related stress as well as loss of services (academic and medical) were associated with the worsening of mental health during the pandemic.  14.0% of participants used acute mental health services during the pandemic.  Gender did not predict mental health deterioration or service use. Age did not predict mental health changes, but older youth were more likely to use acute mental health services. |
| Day et al. (2023)  USA | Autism | Based on self-report, during the pandemic (from June to December 2020), autistic youth had a decrease in some internalizing symptoms (depressive symptoms, generalized anxiety and social anxiety), both overall and relative to NT controls, but no change in separation anxiety, panic disorder or OCD symptoms. NT controls did not experience changes in any of these symptoms.  While there were no differences in average symptoms during the pandemic between groups, both groups demonstrated significant interindividual variability in their responses.  Based on parent-report, during the pandemic, there were no changes in internalizing symptoms in either autistic youth or NT youth.  No findings were presented on gender differences. Age did not predict changes in symptoms. |
| de Maat et al. (2025)  Netherlands | Autism | Compared to NT controls, autistic youth had a higher level of parent-reported internalizing and externalizing problems both before and during the pandemic, but their symptoms remained stable over time.  Although autistic youth and NT youth did not differ in the mean change in symptoms, interindividual variability in responses was higher among autistic youth at both timepoints (the impact of the pandemic was especially heterogeneous in autistic youth).  In the total sample, higher pre-pandemic internalizing and externalizing problems predicted smaller increases in these problems during the pandemic.  Only in NT youth, girls were more likely to have greater increases in internalizing and externalizing problems compared to boys. In autistic youth, gender was not correlated with the symptom change. In both groups, age did not predict symptom change. |
| Gonzales et al. (2023)  USA | Autism | In the first three months of the pandemic, ABA therapy utilization decreased, losing an average of 10.65 hours of therapy per month. In the following nine months, ABA therapy utilization increased, gaining an average of 2.38 hours per month, but did not reach the pre-pandemic levels.  No findings were presented on gender differences. Older youth used less therapy over time compared to younger youth. |
| Hooijman et al. (2024)  Netherlands | Autism | Based on parents’ ratings, challenges associated with autism remained relatively stable over the course of the pandemic, with their severity similar to the pre-pandemic levels.  Pre-pandemic severity predicted the severity during the pandemic. Gender and age did not predict changes in the severity. |
| Lugo-Marín et al. (2021)  Spain | Autism | In the total sample, there was no change in any subscale of symptoms during the pandemic. Yet, those with a higher severity of challenges associated with autism had lower internalizing, rule-breaking and total problems compared to those with the lower severity.  At the same time, those with a higher severity changed their pharmacological treatment (due to worsening of their state) more often than those with a lower severity (27% vs. 15%).  In the survey, 57% and 38% of parents reported that their children experienced a worsening in mood/irritability and anxiety, while 49% of parents noticed improvements in feeding quality.  No findings were presented on gender differences. There were no age differences during the pandemic. |
| Mpaka et al. (2026)  Democratic Republic of Congo | Autism | 42.6% of parents reported a decrease in their children’s challenges associated with autism during the pandemic, while 57.4% reported no change or worsening.  Comorbid conditions (e.g., ID, ADHD) were linked to negative changes in challenges associated with autism during the pandemic.  Gender and age at diagnosis did not predict the outcomes. |
| Palmer et al. (2023)  England | Autism | Based on parents’ ratings, greater pre-pandemic externalizing symptoms (ADHD symptoms) and communication challenges were linked to higher internalizing symptoms during the pandemic.  No association was found between the levels of internalizing symptoms before and during the pandemic.  Age was not associated with symptom severity during the pandemic. No findings were presented on gender differences. |
| Siracusano et al. (2021)  Italy | Autism | Based on parents’ ratings, during the pandemic, there was an improvement in adaptive functioning among preschoolers and no change among school-aged children. In preschoolers, this improvement was not associated with gender, but with the absence of ID and the use of web-delivered behavioral intervention. Those preschoolers who did not receive this intervention during the pandemic had no improvement in adaptive functioning.  In school-aged children, ID and intervention use did not predict the outcomes.  There was no change in repetitive behaviors or internalizing and externalizing symptoms during the pandemic in either age group.  No gender differences were found. |
| Taniguchi et al. (2022)  USA | Autism | Patients who visited the autism clinic in 2019 were more likely to return to the clinic during the pandemic if: they were 13–17 years old; had a comorbid condition (anxiety disorder, ADHD, depression or disruptive behavior); had higher rates of psychotropic prescriptions (antidepressants, ADHD medication, antipsychotics); or used two or more psychiatric medications/psychotropic classes.  Gender did not predict the return to the clinic. |
| Türkoğlu et al. (2021)  Turkey | Autism | Based on parents’ ratings, during the pandemic, autistic youth had worsened irritability, social and self-help skills, as well as sensory and language issues.  Before the pandemic, there were no correlations between irritability and challenges associated with autism; yet, those associations emerged during the pandemic; especially so for social and self-help issues.  No findings were presented on gender or age differences. |
| Vasa et al. (2021)  USA | Autism | Based on parents’ ratings, 53% of youth developed new symptoms during the pandemic, with the most common issues including anxiety, irritability and disruptive behaviors.  In verbal youth, COVID-19 understanding was associated with greater psychiatric problems.  Gender and age did not predict increased psychiatric problems during the pandemic. |
| Breaux et al. (2021)  USA | ADHD | Compared to NT controls, youth with ADHD had a higher likelihood of showing increased levels of parent-reported externalizing symptoms (inattentiveness, hyperactivity/impulsivity and ODD behaviors) during the pandemic. ADHD status (presence/absence) did not predict changes in internalizing symptoms (depression and anxiety).  Compared to youth with ADHD and better pre-pandemic emotion regulation skills, youth with ADHD and poorer emotion regulation skills were reported to have greater inattention and hyperactivity/impulsivity during the pandemic.  In the total sample, boys were more likely to experience an increase in hyperactivity/impulsivity during the pandemic, compared to girls. Gender did not predict any other symptoms. No findings were presented on age differences. |
| Çetin et al. (2020)  Turkey | ADHD | A higher self-reported severity of the psychological impact of the pandemic was correlated with higher parent-reported ODD symptoms but not with ADHD or CD symptoms.  No findings were presented on gender or age differences. |
| Cunniff et al. (2024)  USA | ADHD | During the pandemic, the number of patients refilling their ADHD medication significantly decreased, with fewer refills every pandemic month (31–44%) compared to pre-pandemic months (40–46%), with the only exception of July 2019. Refill rates did not reach baseline when schools reopened in 2021 (they stayed unchanged).  During the pandemic, there was also a decrease in youth attending regular appointments related to symptom management and medication use (from 59–70% to 33–50%).  Refills were higher among youth who attended both online and in-person appointments compared to those with just in-person ones.  Boys who attended in-person and online appointments had higher refill rates than girls with both in-person and online appointments.  Older youth were less likely to refill ADHD prescriptions consistently over time and attended fewer ADHD-related appointments during the pandemic. |
| Kuygun Karci & Arici Gurbuz (2022)  Turkey | ADHD | Based on parents’ ratings, youth who did sports activities prior to the pandemic had lower conduct problems, hyperactivity/impulsivity and inattention during the pandemic.  During the pandemic, 66% stopped taking their pre-existing ADHD medication. 70.6% of those who continued their medications during the pandemic had a psychiatric comorbidity (there was a significant correlation between these variables). There was no difference in the mean symptoms between those who continued and stopped taking their medication.  No findings were presented on gender or age differences. |
| Melegari et al. (2021)  Italy | ADHD | Youth with a low pre-pandemic severity of internalizing and externalizing problems had a significant worsening in these dimensions during the lockdown, according to their parents. Meanwhile, those with a high pre-pandemic severity improved in opposition, restlessness and irritability but worsened in low enjoyment/interest and boredom during the pandemic.  No findings were presented on gender differences. Overall, while children (aged 5–11) and adolescents (aged 12–17) had similar trends of improvement/worsening, children had more deterioration in mood-related problems than adolescents during the pandemic. |
| Uçar et al. (2022)  Turkey | ADHD | Compared to youth with a lower self-reported severity of the psychological impact of the pandemic, youth with a higher severity had greater parent-reported irritability, depression, generalized anxiety, ODD and total ADHD symptoms during the pandemic. Irritability mediated the relationships that the pandemic-related psychological impact, depression and anxiety symptoms had with the total ADHD symptoms.  Age and gender did not predict the severity of the psychological impact of the pandemic. |
| Studies focusing on multiple ND groups | | |
| Suzuki & Hiratani (2021)  Japan | A range of neurodivergences, including ADHD, autism and ID | Based on parents’ ratings, during the pandemic, youth irritability and hyperactivity decreased, while lethargy, stereotypic behaviors and inappropriate speech did not change. Changes in scores during the pandemic did not depend on the type of neurodivergence.  No findings were presented on gender or age differences. |
| Studies focusing on a single pre-existing psychiatric disorder | | |
| Henein et al. (2024)  England | OCD | Based on clinicians’ ratings, 46.7% of youth had a worsening of their symptoms during the pandemic, while 13.3% stayed the same and 40% improved.  No findings were presented on gender or age differences. |
| Nissen et al. (2020)  Denmark | OCD | 44.6% of youth reported a worsening of OCD symptoms during the pandemic, while 36.9% reported no change and 18.5% reported an improvement.  32.3% and 33.8% of youth reported an increase in anxiety and depressive symptoms respectively.  OCD symptom worsening was the most notable among participants who were younger or had an earlier age of OCD onset. Baseline sexual or aggressive obsessive-compulsive thoughts and rituals and pandemic worsening of depressive and anxiety symptoms were also associated with worsened OCD symptom severity during the pandemic.  Gender, baseline severity, comorbidities, receiving psychiatric therapy or taking SRIs were not significant predictors of OCD severity during the pandemic. |
| Schwartz- Lifshitz et al. (2024)  Israel | OCD | Based on clinicians’ ratings, 21% of youth had a worsening of OCD symptoms during the pandemic, while 34% stayed the same and 45% improved.  The mean OCD severity, measured by the clinicians, did not significantly differ across the three timepoints (pre-pandemic, the first wave, two years later). The self-reported level of functioning stayed stable across the two pandemic measurements.  Compared to boys, girls were more likely to take SRIs, which was in turn associated with worse self-reported functioning. Girls were also more likely to have deterioration in their symptoms during the pandemic, according to both self-report and clinicians’ ratings. Comorbidities did not predict OCD symptoms. No findings were presented on age differences. |
| Tanir et al. (2020)  Turkey | OCD | Based on clinicians’ ratings, there was an increase in obsessions, compulsions and severity of OCD symptoms during the pandemic, with 54.1% of youth experiencing a worsening of OCD symptoms. 31.1% of youth who had a remission before the pandemic relapsed during the pandemic. Fewer youth received medicated treatment during the pandemic.  This worsening was the most notable among those with the longer duration of OCD diagnosis and those who were regularly worried about the pandemic or exposed to pandemic-related content through their environment or the media.  No findings were presented on gender or age differences. |
| Akgül et al. (2022)  Turkey | ED | During the pandemic, 42.1% of youth reported an improvement in their ED symptoms, while 21.1% reported a worsening; yet, only 39.5% reported regular meal plan compliance during the pandemic.  23.7% felt that the pandemic often negatively affected their access to the relevant healthcare services.  Depressive symptoms during the pandemic were the strongest predictor of ED behaviors. While only 7.9% had depression before the pandemic, 52.6% exceeded the clinical cut-off during the pandemic.  No findings were presented on gender or age differences. |
| Torun et al. (2024)  Turkey | ED | *This study is a follow-up for Akgül et al. (2021).* Participants with pre-existing EDs were assessed at the beginning of the pandemic (Akgül et al., 2021) and then one year later (current study).  Participants’ BMI had increased at the start of the pandemic but did not change significantly a year into the pandemic. A year into the pandemic, the target weight percentage was higher than before the pandemic, but not higher than earlier stages of the pandemic.  There was no significant change in self-reported ED behavior or diet compliance compared to the first pandemic observation. Self-reported OCD and depressive symptoms remained stable a year into the pandemic while anxiety significantly increased.  The proportion of participants who often had difficulty in accessing ED healthcare decreased, reaching 2.7%. The percentages of youth using psychotropic medication stayed stable across two pandemic stages.  Depression consistently played the largest role in predicting ED behavior at both the early and later stages, while anxiety became the second most significant predictor in the later stage. Age was not correlated with anxiety, depression, OCD or ED symptoms during the pandemic.  No findings were presented on gender differences. |
| Kuz et al. (2023)  Turkey | Anxiety disorders | Compared to youth with a lower self-reported severity of the psychological impact of the pandemic, youth with a higher severity had greater self-reported emotion regulation problems during the pandemic. A higher self-reported severity of the psychological impact of the pandemic was also correlated with greater parent-reported anxiety.  Compared to boys, girls were more likely to experience severe psychological stress during the pandemic. No age differences were detected. |
| Sadeghi et al. (2022)  USA | MDD | During the pandemic, the severity of depressive and anxiety symptoms slightly decreased in youth with MDD, compared to their pre-pandemic levels. Among healthy controls, depressive symptoms increased during the pandemic; yet, their scores were still significantly lower than those of youth with depression. Findings were similar across informants.  In youth with MDD, the decrease in self-reported depressive symptoms during the pandemic was significant only in boys and older youth (girls and younger youth had no significant change). Similarly, in healthy controls, the increase in self-reported symptoms was significant only in girls and younger youth (boys and older youth had no significant change).  Based on the survey, youth with MDD had greater self-reported worries related to the pandemic compared to controls. |
| De Nardi et al. (2021)  Italy | SSD | During the pandemic, youth with SSD reported having fewer depressive and anxiety symptoms, compared to the healthy control group without SSD. While most youth received psychological treatment before the pandemic, they did not have it during the pandemic.  No findings were presented on gender or age differences. |
| Studies focusing on multiple types of pre-existing conditions | | |
| Bilu et al. (2023)  Israel | Neurodivergences (ADHD); a range of psychiatric disorders, including depression, anxiety, ED, OCD and stress-related disorders | During the pandemic, ND youth and youth with previous psychiatric histories had an increase (+9%) in antidepressants use and a decline (-13%) in the use of ADHD medication (i.e., psychostimulants and nootropics). These changes were seen in both boys and girls. There were no statistically significant changes in the use of antipsychotics and anxiolytics.  Meanwhile, NT youth without prior psychiatric conditions saw increases in the incident use of antidepressants (+25%) and antipsychotics (+28%), while ADHD prescriptions (-10%) declined. The increases were only seen in girls. There was no statistically significant change in the anxiolytics use.  Overall, increases were more pronounced among youth aged 14 to 15 and among those without pre-existing conditions. |
| Bliddal et al. (2023)  Denmark | Neurodivergences (autism); a range of psychiatric disorders, including psychotic, mood, anxiety and hyperkinetic disorders | During the pandemic, there was an increase in the use of antidepressants, hypnotics and psychostimulants and no change in the use of anxiolytics, among both ND youth/youth with pre-existing psychiatric disorders and NT youth without prior psychiatric disorders. The use of antipsychotics increased only in youth without prior conditions.  The increase was especially noticeable among those aged 12 to 17 years.  No findings were presented on gender differences. |
| Burrell et al. (2023)  USA | Neurodivergences (ADHD); a range of psychiatric disorders, including anxiety, mood disorders, dual diagnoses of anxiety and mood disorders, psychosis and multiple conditions | During the pandemic, the overall odds of using outpatient mental health services increased by 12% among ND youth and youth with pre-existing psychiatric disorders.  Mental health service utilization increased among youth with a prior anxiety disorder, mood disorder and dual anxiety and mood disorder and decreased for those with ADHD and those with a prior psychosis or multiple conditions.  Before the pandemic, adolescents aged 12–13.9 years were less likely to use mental health services compared to those aged 16–17.9 years; yet, during the COVID-19 period, the likelihood of service use among younger adolescents increased significantly, with odds ratios rising by 36% for those aged 12–13.9 years and by 26% for those aged 14–15.9 years.  During the pandemic, the odds ratio of girls using services compared to boys increased by 63%. Service utilization was higher among girls than boys both before and during the pandemic. |
| Conti et al. (2020)  Italy | Neurodivergences (e.g., autism, ADHD, ID); emotional and behavioral disorders (OCD, anxiety, mood and feeding disorders) | Based on parents’ ratings, during the pandemic, there was an increase in anxiety in preschoolers, and an increase in thought problems and OCD symptoms among school-aged children.  While younger age was a protective factor for preschoolers (linked to decreased total problems and ADHD symptoms), younger age was a risk factor for school-aged children (linked to increased affective problems).  Among school-aged children, those with emotional and behavioral disorders had more symptom improvement during the pandemic than ND youth. In both age groups, parent-reported weekly hours of pre-pandemic treatment were not associated with the symptom change during the pandemic.  No findings were presented on gender differences. |
| Erez et al. (2022)  Israel | Neurodivergences (ADHD); a range of psychiatric disorders, including anxiety, mood, conduct and stress-related disorders | During the pandemic, there was a decrease in the total number of psychiatric emergency visits among youth in general, but an increase in the number of visits among ND youth and youth with pre-existing psychiatric disorders. Compared to pre-pandemic times, during the pandemic, youth with a psychiatric emergency were more likely to have pre-existing conditions.  No findings were presented on gender or age differences. |
| Gilsbach et al. (2021)  Germany | Neurodivergences (ADHD); a range of psychiatric disorders, including depression, anxiety disorders, ED, OCD and PTSD | Compared to NT controls without prior psychiatric disorders, ND youth and youth with pre-existing psychiatric disorders had higher distress associated with the pandemic (according to both self- and parent-report).  Based on self-report, this distress was higher among youth with depressive disorders compared to those with ADHD.  Among ND youth and youth with pre-existing psychiatric disorders, girls had higher self-reported (but not parent-reported) distress, compared to boys. Age was not a significant predictor. |
| Haller et al. (2024)  USA | Neurodivergences (ADHD); a range of psychiatric disorders, including DMDD, ODD and anxiety disorders | During the pandemic, self-reported anxiety significantly increased in both ND youth/those with pre-existing psychiatric disorders and NT controls without prior psychiatric disorders. Increases in self-reported anxiety were similar and slightly larger in controls.  The increase in self-reported depressive symptoms approached significance in both groups (.06–.07).  Changes in parent-reported symptoms were not significant, except for the increase in anxiety in the controls.  There was an association between pre-pandemic and pandemic levels of depressive and anxiety symptoms across both types of informants. Age was not associated with the symptom levels during the pandemic. No findings were presented on gender differences. |
| Khan et al. (2022)  Qatar | Neurodivergences; a range of psychiatric disorders, including OCD, mood, anxiety and conduct disorders | 90.5% of youth reported having high COVID-related worries. Each diagnostic group had significant OCD symptoms during the pandemic. Moreover, the “mood and anxiety disorders” group had a higher mean score than the OCD group.  In the total sample, pandemic-related worrying was associated with greater OCD symptoms. This finding was not limited to youth with an OCD diagnosis. The type of intervention was not associated with worrying or OCD symptoms during the pandemic.  No gender or age differences were detected. |
| Lewis et al. (2023)  USA | Neurodivergences (ADHD) and internalizing disorders (anxiety and depressive disorders) | Compared to youth without internalizing disorders, youth with internalizing disorders were more likely to report moderate or clinically significant psychological trauma scores related to the pandemic. Meanwhile, ADHD did not predict the level of trauma during the pandemic.  In the total sample, throughout the pandemic, girls had higher levels of trauma and were more likely to exceed the clinical threshold for it, compared to boys.  No findings were presented on age differences. |
| Lund et al. (2025)  Norway | Neurodivergences (ADHD); a range of psychiatric disorders, including anxiety and depression | During the pandemic, there was a decrease in the weekly number of psychiatric consultations among youth with depression, anxiety or ADHD, compared to pre-pandemic times. This decrease was more pronounced: in youth with depression or anxiety, compared to those with ADHD; during later pandemic periods; and among boys, compared to girls.  No findings were presented on age differences. |
| Operto, Coppola, et al. (2022)  Italy | Neurodivergences (autism, ADHD, ID); a range of psychiatric disorders, including anxiety, mood and behavioral disorders | Based on parents’ ratings, during the pandemic, there was an increase in internalizing and externalizing symptoms in autistic youth (both younger and older youth), youth with ADHD, anxiety, mood and behavioral disorders. Youth with ID also had increases in these symptoms, except for withdrawal, emotional reactivity and attention problems.  During the survey, 43.9% of parents reported that their child’s condition worsened during the pandemic. 28.2% reported that their child developed eating problems that were absent before.  In the total sample, all symptoms increased in both age groups (1.5–5 and 6–18). The effect sizes were higher in school-aged youth, with the exception of aggression which increased more in preschoolers.  No findings were presented on gender differences. |
| Operto, Scaffidi Abbate, et al. (2022)  Italy | Neurodivergences (autism, ADHD, ID); a range of psychiatric disorders, including anxiety, mood and behavioral disorders | Based on parents’ ratings, during the pandemic, there was an increase in internalizing and externalizing symptoms in autistic youth, youth with ID, anxiety, mood and behavioral disorders. Meanwhile, youth with ADHD only demonstrated an increase in somatic complaints (part of internalizing symptoms).  In contrast to the type of pre-existing condition, gender and age did not predict the outcomes. |
| Parlatini et al. (2024)  England | Neurodivergences (autism, ADHD, ID) and emotional disorders (depression, anxiety disorders, PTSD and OCD) | Based on parents’ ratings, during the pandemic, autistic youth and youth with ADHD experienced a greater worsening of internalizing and externalizing symptoms than those with emotional disorders.  Autistic youth experienced a greater worsening of internalizing symptoms and a higher level of pandemic-related worries compared to youth with ADHD. Having multiple neurodivergences (i.e., autism and ADHD) was associated with greater internalizing and externalizing symptoms during the pandemic. Having a comorbid ID predicted fewer internalizing symptoms.  36.2% and 32% of parents reported declines in quality and quantity of youth mental health services. In the total sample, service dissatisfaction predicted greater internalizing and externalizing symptoms among youth during the pandemic; girls and older youth were more likely to have internalizing problems during the pandemic than boys and younger youth. Being younger was associated with more externalizing problems. |
| Payne et al. (2023)  Australia | Neurodivergences (ADHD); a range of psychiatric disorders, including mood, anxiety, ED, OCD and stress-related disorders | During the pandemic, 52.2% of parents reported a worsening of their children’s symptoms. Attending school remotely was linked to symptom worsening, higher sadness and less focus. Completing the survey during the easing of COVID-related restrictions was also associated with symptom worsening, as well as with higher anxiety, less enjoyment and focus.  Gender did not predict changes in these symptoms, while older age was associated with more sadness, social issues and symptom worsening.  Nearly a third of parents indicated that the quality (28.5%) and quantity (30.3%) of mental health care for their child declined compared to pre-pandemic levels. However, 47.7% still felt that the current services were adequately meeting their needs. |
| Şentürk Pilan et al. (2021)  Turkey | Neurodivergences (ADHD, ID); a range of psychiatric disorders, including anxiety disorders, psychosis and PTSD | The severity of the cases, measured by the clinicians, increased during the pandemic.  26% of youth mentioned an increase in anxiety during the pandemic; 39.2% did not notice any change in their symptoms.  No findings were presented on gender or age differences. |
| Şentürk Pilan et al. (2023)  Turkey | Neurodivergences (autism, ADHD); a range of psychiatric disorders, including MDD, anxiety disorders, conduct disorders and OCD | During the early stages of the pandemic, no changes in the clinician-rated symptom severity were found for youth with a single pre-existing condition; meanwhile, youth with multiple conditions had an improvement in their functioning.  Throughout the later stages of the pandemic, neither group exhibited significant changes.  Youth with multiple conditions were less satisfied with the provided mental health services than youth with a single condition, and lower satisfaction was linked to higher symptom severity during the early stages of the pandemic.  No findings were presented on gender or age differences. |
| Shankar et al. (2022)  USA | A range of psychiatric disorders (unspecified) | During the pandemic, there was an increase in the proportion of psychiatric emergency visits by youth with pre-existing psychiatric disorders.  No findings were presented on gender or age differences. |
| Stevanovic et al. (2022)  Georgia, Lithuania, Romania, Serbia and Turkey | Neurodivergences (autism, ADHD); a range of psychiatric disorders, including anxiety, mood, OCD, psychotic and conduct disorders | 49.8% of parents reported that their children experienced a worsening of their main pre-existing symptoms during the pandemic, 26.3% noticed some improvement, while 23.9% saw no changes.  33.1% and 43.1% of parents reported deterioration in quality and quantity of youth mental health services. Based on parental reports, reduced access to mental health care and youth COVID-related worries significantly predicted youth internalizing problems.  Based on youth’ ratings, 43.9% and 40.6% had a worsening in internalizing and externalizing symptoms respectively; 18.1% and 17.9% noticed some improvement; while 38% and 41.6% reported that these symptoms did not change.  Based on self-reports, those with greater worries and girls had a greater risk of having internalizing problems during the pandemic; a lack of satisfaction about the received mental health care predicted both internalizing and externalizing problems.  No findings were presented on age differences. |
| Zijlmans et al. (2021)  Netherlands | Neurodivergences (autism, ADHD); a range of psychiatric disorders (e.g., depression) | Compared to NT controls without prior psychiatric disorders, ND youth and youth with pre-existing psychiatric disorders reported having worse overall mental health, as well as greater anger and depressive symptoms during the pandemic.  Among ND youth and youth with pre-existing psychiatric disorders, older age predicted worse mental health and lower anger. Boys had better mental health and lower depressive symptoms, compared to girls. |

*Note.* ABA = applied behavior analysis; ADHD = attention-deficit/hyperactivity disorder; BMI = body mass index; DMDD = disruptive mood dysregulation disorder; ED = eating disorder; ID = intellectual disability; MDD = major depressive disorder; ND = neurodivergent; NT = neurotypical; OCD = obsessive-compulsive disorder; ODD = oppositional defiant disorder; PTSD = post-traumatic stress disorder; SSD = somatic symptom disorder; SRIs = serotonin reuptake inhibitors; USA = United States of America.

**Table S2**

*Youth-Related Factors Linked to Mental Health Outcomes and Psychiatric Healthcare Utilization Across Included Studies*

| Factor | Studies | *n* of studies |
| --- | --- | --- |
| Age | Berard et al., 2021; Bilu et al., 2023; Bliddal et al., 2023; Burrell et al., 2023; Charalampopoulou et al., 2022; Conti et al., 2020; Gonzales et al., 2023; Melegari et al., 2021; Nissen et al., 2020; Operto, Coppola, et al., 2022; Parlatini et al., 2024; Payne et al., 2023; Sadeghi et al., 2022; Siracusano et al., 2021; Taniguchi et al., 2022; Zijlmans et al., 2021 | 16 |
| Gender | Breaux et al., 2021; Burrell et al., 2023; Cunniff et al., 2024; Gilsbach et al., 2021; Kuz et al., 2023; Lewis et al., 2023; Lund et al., 2025; Parlatini et al., 2024; Sadeghi et al., 2022; Schwartz-Lifshitz et al., 2024; Stevanovic et al., 2022; Zijlmans et al., 2021 | 12 |
| Pre-pandemic condition | Burrell et al., 2023; Conti et al., 2020; Gilsbach et al., 2021; Khan et al., 2022; Lewis et al., 2023; Lund et al., 2025; Operto, Coppola, et al., 2022; Operto, Scaffidi Abbate, et al., 2022; Parlatini et al., 2024 | 9 |
| Access to healthcare | Berard et al., 2021; Charalampopoulou et al., 2022; Cunniff et al., 2024; Parlatini et al., 2024; Şentürk Pilan et al., 2023; Siracusano et al., 2021; Stevanovic et al., 2022 | 7 |
| Comorbid conditions | Burrell et al., 2023; Kuygun Karci & Arici Gurbuz, 2022; Mpaka et al., 2026; Parlatini et al., 2024; Şentürk Pilan et al., 2023; Siracusano et al., 2021; Taniguchi et al., 2022 | 7 |
| COVID-related stress and worries | Çetin et al., 2020; Charalampopoulou et al., 2022; Khan et al., 2022; Kuz et al., 2023; Stevanovic et al., 2022; Tanir et al., 2020; Uçar et al., 2022 | 7 |
| Pre-pandemic symptom severity | Charalampopoulou et al., 2022; de Maat et al., 2025; Haller et al., 2024; Hooijman et al., 2024; Lugo-Marin et al., 2021; Melegari et al., 2021; Palmer et al., 2023 | 7 |
| Emotion regulation skills | Breaux et al., 2021; Kuz et al., 2023 | 2 |
| Irritability | Türkoğlu et al., 2021; Uçar et al., 2022 | 2 |

**Table S3**

*Number of Studies Per Country*

| **Country** | ***n* of studies** |
| --- | --- |
| USA | 11 |
| Turkey | 10 |
| Italy | 6 |
| England | 3 |
| Israel | 3 |
| Netherlands | 3 |
| Denmark | 2 |
| Australia | 1 |
| Canada | 1 |
| Democratic Republic of Congo | 1 |
| France | 1 |
| Germany | 1 |
| Japan | 1 |
| Norway | 1 |
| Qatar | 1 |
| Spain | 1 |
| Multiple countries | 1 |

**Table S4**

*Risk of Bias Ratings Across Included Studies*

| **Authors** | **Recruitment/ sampling** | **Sample size** | **Outcome assessment** | **Attrition** | **Confounding** | **Overall rating** |
| --- | --- | --- | --- | --- | --- | --- |
| Studies focusing on a single neurodivergent group | | | | | | |
| Autism | | | | | | |
| Berard et al. | High | Moderate | Low | High | Low | Moderate |
| Charalampopoulou et al. | High | Moderate | Moderate | High | Low | Moderate |
| Day et al. | Moderate | Moderate | Moderate | High | Moderate | Moderate |
| de Maat et al. | Moderate | Moderate | Moderate | Low | Moderate | Moderate |
| ***Gonzales et al.*** | Moderate | Moderate | Low | N/A | Moderate | Moderate |
| Hooijman et al. | Moderate | Moderate | Moderate | High | Moderate | Moderate |
| Lugo-Marin et al. | High | High | Moderate | Moderate | Moderate | High |
| Mpaka et al. | High | Moderate | High | Moderate | Low | Moderate |
| Palmer et al. | Moderate | High | Moderate | Moderate | Moderate | Moderate |
| Siracusano et al. | High | Moderate | Moderate | Moderate | Moderate | Moderate |
| ***Taniguchi et al.*** | Moderate | High | Low | N/A | Moderate | Moderate |
| Türkoğlu et al. | High | Moderate | Moderate | Moderate | High | High |
| Vasa et al. | High | Moderate | High | High | Low | Moderate |
| Attention-deficit/hyperactivity disorder | | | | | | |
| Breaux et al. | High | Low | Moderate | Low | Low | Moderate |
| Çetin et al. | High | High | Moderate | Moderate | High | High |
| ***Cunniff et al.*** | Moderate | High | Low | N/A | Moderate | Moderate |
| Kuygun Karci & Arici Gurbuz | High | High | Moderate | High | Moderate | High |
| Melegari et al. | Moderate | Moderate | Moderate | High | Moderate | Moderate |
| Uçar et al. | High | High | Moderate | Moderate | Low | Moderate |
| Studies focusing on multiple neurodivergent groups | | | | | | |
| Suzuki & Hiratani | Moderate | Moderate | Moderate | High | Moderate | Moderate |
| Studies focusing on a single pre-existing psychiatric disorder | | | | | | |
| Obsessive-compulsive disorder | | | | | | |
| ***Henein et al.*** | High | High | Low | N/A | High | Moderate |
| Nissen et al. | High | High | Low | Moderate | Low | Moderate |
| Schwartz-Lifshitz et al. | Moderate | High | Low | Moderate | Moderate | Moderate |
| Tanir et al. | High | Moderate | Low | Moderate | Moderate | Moderate |
| Eating disorders | | | | | | |
| Akgül et al. | High | Moderate | Moderate | Moderate | Moderate | Moderate |
| Torun et al. | High | High | Low | Low | Moderate | Moderate |
| Anxiety disorders | | | | | | |
| Kuz et al. | High | High | Moderate | Moderate | Moderate | High |
| Major depressive disorder | | | | | | |
| Sadeghi et al. | Moderate | Moderate | Moderate | Low | Moderate | Moderate |
| Somatic symptom disorder | | | | | | |
| De Nardi et al. | High | High | Moderate | Moderate | High | High |
| Studies focusing on multiple types of pre-existing conditions | | | | | | |
| ***Bilu et al.*** | Low | Low | Low | N/A | Low | Low |
| ***Bliddal et al.*** | Low | Low | Low | N/A | Moderate | Moderate |
| ***Burrell et al.*** | Moderate | Moderate | Low | N/A | Low | Moderate |
| Conti et al. | High | High | Moderate | High | Moderate | High |
| ***Erez et al.*** | Moderate | Moderate | Low | N/A | High | Moderate |
| Gilsbach et al. | High | Low | Moderate | High | Low | Moderate |
| Haller et al. | High | Moderate | Moderate | Moderate | Moderate | Moderate |
| Khan et al. | High | High | Moderate | Moderate | Low | Moderate |
| Lewis et al. | High | Moderate | Moderate | Moderate | Moderate | Moderate |
| ***Lund et al.*** | Low | Moderate | Low | N/A | Moderate | Moderate |
| Operto, Coppola, et al. | High | Moderate | Moderate | Low | Moderate | Moderate |
| Operto, Scaffidi Abbate, et al. | High | Moderate | Moderate | Low | Low | Moderate |
| Parlatini et al. | Moderate | Low | High | Moderate | Low | Moderate |
| Payne et al. | Moderate | High | Moderate | High | Low | Moderate |
| Şentürk Pilan et al. (2021) | High | High | Low | Moderate | High | Moderate |
| Şentürk Pilan et al. (2023) | High | High | Low | Moderate | Moderate | Moderate |
| ***Shankar et al.*** | Moderate | Moderate | Low | N/A | High | Moderate |
| Stevanovic et al. | High | Moderate | High | High | Low | Moderate |
| Zijlmans et al. | Moderate | Moderate | Moderate | High | Low | Moderate |

*Note*. Lower ratings signified a lower risk of bias. Register-based studies were put in bold and italics. All register-based studies had N/A (not applicable) ratings for attrition.

**References**

Ching, B. C. F., Downs, J., Zhang, S., Abdul Cader, H., Penhallow, J., Voraite, E., Popnikolova, T., Wickersham, A., Parlatini, V., & Simonoff, E. (2025). Research review: The impact of the COVID‐19 pandemic on the mental health of children and young people with pre‐existing mental health and neurodevelopmental conditions – A systematic review and meta‐analysis of longitudinal studies. *Journal of Child Psychology and Psychiatry*, *66(*8), 1274–1298. https://doi.org/10.1111/jcpp.14117

Cost, K. T., Crosbie, J., Anagnostou, E., Birken, C. S., Charach, A., Monga, S., ... & Korczak, D. J. (2022). Mostly worse, occasionally better: Impact of COVID-19 pandemic on the mental health of Canadian children and adolescents. *European Child & Adolescent Psychiatry*, *31*(4), 671–684. https://doi.org/10.1007/s00787-021-01744-3

Kroon, R., Bothma, N., Mathieu, S., Fontenelle, L. F., & Farrell, L. J. (2022). Parental surveillance of OCD and mental health symptoms during COVID-19: A longitudinal study of Australian children, adolescents and families. *Journal of Psychiatric Research*, *152*, 225–232. https://doi.org/10.1016/j.jpsychires.2022.06.002

Sicouri, G., March, S., Pellicano, E., De Young, A. C., Donovan, C. L., Cobham, V. E., Rowe, A., Brett, S., Russell, J. K., Uhlmann, L., & Hudson, J. L. (2023). Mental health symptoms in children and adolescents during COVID-19 in Australia. *Australian & New Zealand Journal of Psychiatry*, *57(*2), 213–229. https://doi.org/10.1177/00048674221090174
